# Malleable, Ultrastrong Antibacterial Thermosets Enabled by Guanidine Urea Structure

**DOI:** 10.1002/advs.202402891

**Published:** 2024-06-13

**Authors:** Zhen Yu, Qiong Li, Yanlin Liu, Shu Tian, Wanding Chen, Yingying Han, Zhaobin Tang, Junping Zhang

**Affiliations:** ^1^ Center of Eco‐Material and Green Chemistry Lanzhou Institute of Chemical Physics Chinese Academy of Sciences Lanzhou 730000 P. R. China; ^2^ University of Chinese Academy of Sciences Beijing 100049 P. R. China; ^3^ Department of Chemistry The University of Hong Kong Hong Kong 999077 P. R. China; ^4^ Ningbo Institute of Materials Technology and Engineering Chinese Academy of Sciences Ningbo 315201 P. R. China

**Keywords:** antibacterial, dynamic covalent polymer, guanidine urea, recycling, supramolecular

## Abstract

Dynamic covalent polymers (DCPs) that strike a balance between high performance and rapid reconfiguration have been a challenging task. For this purpose, a solution is proposed in the form of a new dynamic covalent supramolecular motif—guanidine urea structure (GUAs). GUAs contain complex and diverse chemical structures as well as unique bonding characteristics, allowing guanidine urea supramolecular polymers to demonstrate advanced physical properties. Noncovalent interaction aggregates (NIAs) have been confirmed to form in GUA‐DCPs through multistage H‐bonding and π‐π stacking, resulting in an extremely high Young's modulus of 14 GPa, suggesting remarkable mechanical strength. Additionally, guanamine urea linkages in GUAs, a new type of dynamic covalent bond, provide resins with excellent malleability and reprocessability. Guanamine urea metathesis is validated using small molecule model compounds, and the temperature dependent infrared and rheological behavior of GUA‐DCPs following the dissociative exchange mechanism. Moreover, the inherent photodynamic antibacterial properties are extensively verified by antibacterial experiments. Even after undergoing three reprocessing cycles, the antibacterial rate of GUA‐DCPs remains above 99% after 24 h, highlighting their long‐lasting antibacterial effectiveness. GUA‐DCPs with dynamic nature, tuneable composition, and unique combination of properties make them promising candidates for various technological advancements.

## Graphical abstract

A new dynamic covalent supramolecular motif, guanidine urea structure, is proposed and exploited to prepare thermosetting materials with reprocessability, ultrahigh modulus, and intrinsic photodynamic antibacterial characteristics. Dynamic exchange reaction of guanamine urea bonds follows the dissociative exchange mechanism. The material's ultrahigh modulus and photodynamic antibacterial characteristics are attributed to noncovalent interaction aggregates caused by multilevel H‐bonding and π‐π stacking.

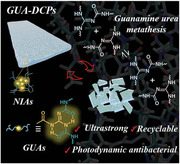

## 1 Introduction

Thermosets exhibit robust mechanical properties and structural integrity that enable these materials to maintain performance under extreme operational and environmental conditions. However, their non‐recyclable nature leads to the generation of millions of tons of waste annually, posing substantial threats to the environment. Polymers with dynamic covalent bonds can undergo reversible chemical reactions, rendering them recyclable and self‐healing. These materials have the potential to avoid wasting by enabling disassembly, repair, and upgrading, thus providing an innovative alternative to traditional polymer.^[^ ^1^ ^]^ Moreover, dynamic covalent bonds facilitate shaping and reshaping of the materials, enabling the production of adaptive and responsive materials with conventional thermoset materials prosperities.^[^ ^2^ ^]^ Although numerous dynamic chemical bonds have been identified to date, such as ester exchange,^[^ ^3^ ^]^ imine bonds,^[^ ^4^ ^]^ acetal bonds,^[^ ^5^ ^]^ disulfide bonds,^[^ ^6^ ^]^ olefin metathesis,^[^ ^7^ ^]^ the Diels‐Alder reaction,^[^ ^8^ ^]^ etc., there remains a trade‐off between macroscopic physical properties and the ability to rebuild networks.^[^ ^9^ ^]^

One considering is to design or improve dynamic motifs that provide reversible crosslinking while ensuring the integrity of the polymer network structure. The combination of fast cure time, durability, chemical resistance, and flexibility makes polyurea materials highly advantageous for various applications, including protective coatings, waterproofing, flooring, corrosion protection, and more.^[^ ^10^ ^]^ Traditional polyurea formation typically requires a catalyst to facilitate the reaction between isocyanate and amine components. In many dynamic chemistry systems, dynamic urea chemistry without any catalysts or additives is a notable dynamic motif that facilitates reversible crosslinking in some cases while maintaining the integrity of the polymer network structure. Moreover, the stability of the urea bond can be affected by the chemical environment, and the introduction of functional groups or conjugated structures on the nitrogen atoms of the urea can induce a decline in the stability of the bond,^[^ ^11^ ^]^ which would result in reversible dissociation or hydrolysis to isocyanates and amines under certain environmental conditions. However, many amine‐derived chemicals that can undergo dynamic polyurea chemistry may not be commercially mainstream, and their complex synthesis pathways can limit their feasibility for widespread use. Additionally, the flexibility and mechanical instability of network structures can also have an impact on the overall durability and performance of the materials and may require careful design considerations to ensure that the properties of the material match its intended application.

Another potential approach involves exploring noncovalent bonds to fine‐tune the balance between the stability of dynamic covalent networks and the mechanical prosperities of the materials.^[^ ^12^ ^]^ Noncovalent interactions such as H‐bonding,^[^ ^13^ ^]^ electrostatic interactions,^[^ ^14^ ^]^ and π‐π stacking^[^ ^15^ ^]^ and so on, can have a significant impact on the macroscopic properties of materials, especially after structural clustering and conformation hardening.^[^ ^16^ ^]^ In a typical example, hydrogen bonds crosslinked composite plastic made of polyacrylic acid and polyvinylpyrrolidone exhibited exceptional mechanical properties due to hydrogen bond aggregates.^[^ ^17^ ^]^ The composite material showed a tensile strength of over 80 MPa and a Young's modulus of over 4.5 GPa. These impressive mechanical properties are challenging to achieve for most dynamic covalent networks, highlighting the potential of noncovalent interactions like hydrogen bonding in improving the performance of polymer materials.

The development of antibacterial plastics presents a promising avenue for combating microbial contamination in various applications. Thermosets offer versatility in terms of shaping and application. They can be molded into various forms, such as films, containers, or surfaces, allowing for a wide range of antibacterial applications. Its flexibility and durability are particularly advantageous in industries like healthcare, where different products and surfaces require perennial antibacterial protection. In many cases, the integration of functional components to impart antibacterial activity may inadvertently impact the performance of materials in terms of thermal stability, mechanical properties, durability, and appearance.^[^ ^18^ ^]^ Therefore, achieving effective antimicrobial functionality without compromising other essential properties poses a significant challenge.

**Scheme 1:**
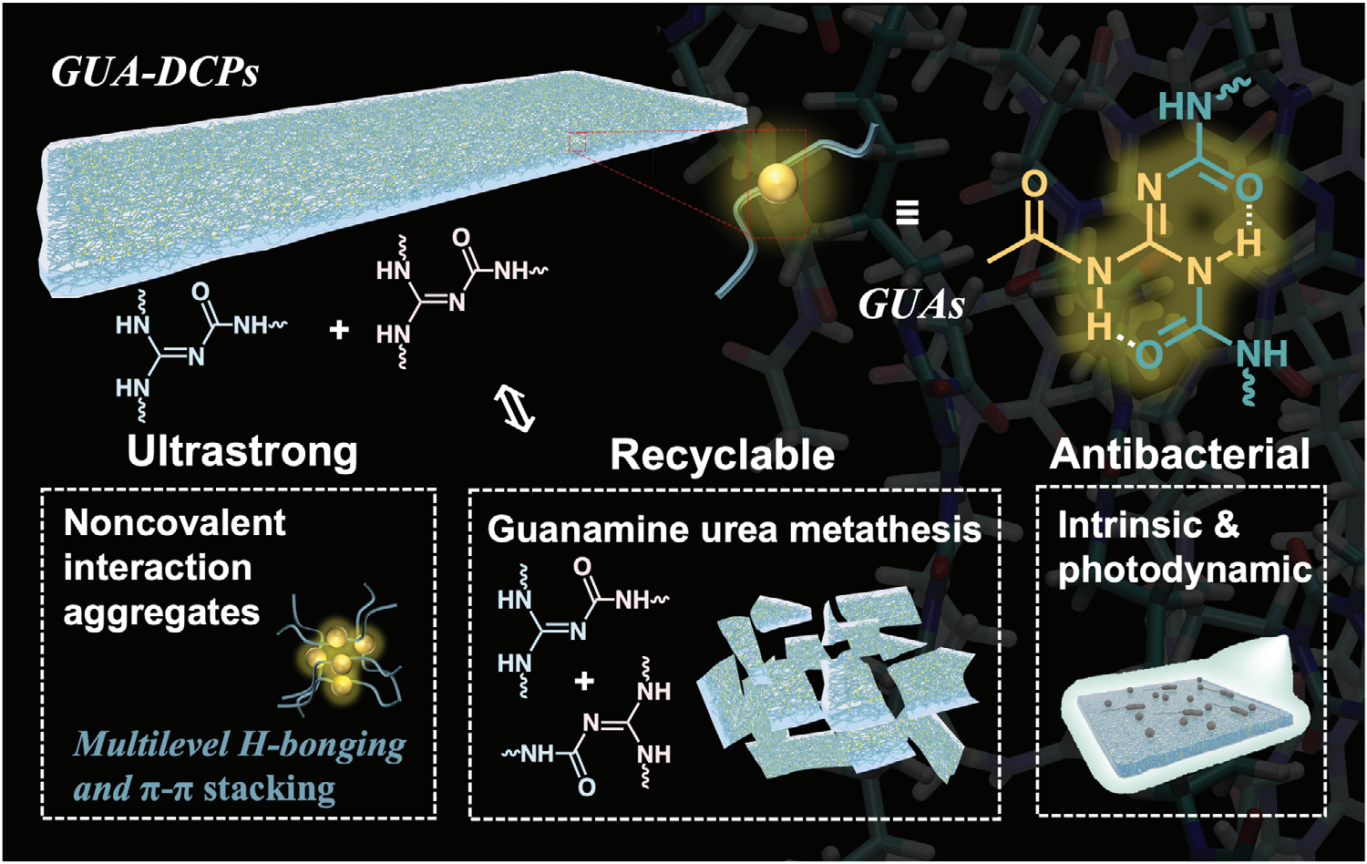
Schematic representation of the GUAs and derived GUA‐DCPs with superior properties.

Herein, we came up with a new dynamic covalent supramolecular guanidine urea structure (GUAs) and constructed guanidine urea supramolecular polymer networks (**Scheme** **1**). GUAs are highly complex and diverse, which gives the resulting GUA‐dynamic covalent polymers (DCPs) multiple significant properties. The building of supramolecular networks is driven by multistage H‐bond structures and π‐π packing, which were experimentally verified using model compounds and in‐situ FTIR spectra. Molecular dynamics (MD) simulations were also used to simulate the formation of the networks. The presence of multiple and aggregated weak interactions in GUA‐DCPs leads to the formation of aggregates, resulting in a high modulus of up to 14 GPa. Additionally, the dynamic exchange reaction of the guanamine urea bonds in GUAs was verified using model compounds. The activation energy of the bond exchange was found to be 47.5 ± 6.2 kJ mol^−1^, and the material could be reprocessed by hot pressing at 140 °C and 10 MPa for 5 min. Furthermore, GUA‐DCPs exhibited a high bacteriostatic rate against *E. coli* and *S. aureus* thanking to imine bonds in GUAs. The chromophore clusters in networks also demonstrated efficient photodynamic antibacterial activity. The antibacterial efficiency of the GUA‐DCPs remained high even after reprocessing, with the antibacterial rate of *E. coli* still being over 99% after 24 h following three rounds of processing.

## 2 Results and Discussion

### 2.1 Fabrication and Characterization of PGUAs

The chemistry of imine compounds has been an active area of research for nearly two centuries since the discovery of the “Schiff bond” by German chemist Hugo Schiff in 1864.^[^ ^19^ ^]^ Acyl hydrazone bonds,^[^ ^20^ ^]^ as a significant branch of imine chemistry, have garnered considerable attention from scientists. One significant factor driving interest in acyl hydrazone bonds is their ability to form strong H‐bonding, similarity to amide bonds, which can provide benefits in various applications. Based on the foregoing, the development of a novel and unique imine urea chemistry that resembles acyl hydrazone has garnered our significant interests and efforts. However, the synthesis of imine urea bonds presents a unique challenge compared to traditional imine chemistry, particularly in the context of urea formaldehyde condensation reactions. In the urea formaldehyde condensation reaction, the formation of imine urea bonds is generally not observed. Instead, the reaction predominantly leads to the formation of alkylidene or arylene diamine (Scheme S1 and Figure S1, Supporting Information). This limitation has motivated us to look for alternative methods for the synthesis of imine urea bonds. Ultimately, we successfully obtained GUAs containing imine urea bonds by a method involving the addition polymerization of guanidine groups and isocyanates (Scheme S2 and Figures S2 and S3, Supporting Information). Guanidine compounds contain the functional group *C = NH* and are used as mainly starting materials in the synthesis of urea imine. This compound is obtained through non‐traditional imine chemistry where the urea imine bond is named as guanamine urea. This method differs from traditional imine chemistry in that it does not require the addition of catalysts and typically does not generate small molecules as byproducts. This feature is advantageous as it simplifies the synthetic pathway and offers potential benefits in terms of atom efficiency and waste reduction.

**Figure 1:**
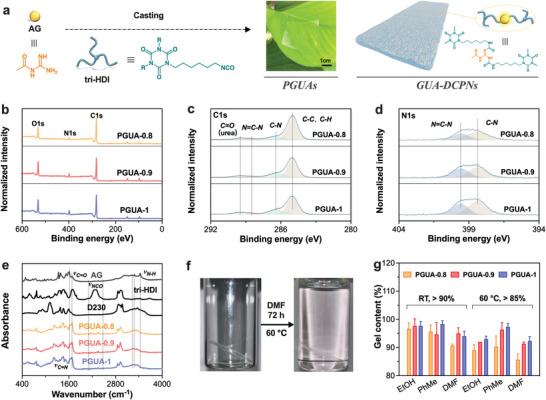
Fabrication of PGUAs. a) Preparation route of PGUAs and the schematic diagram of its crosslinking network; XPS spectra of PGUA‐0.8, PGUA‐0.9, and PGUA‐1 showing b) wide spectrum (0‐600 eV), c) C1s (280‐292 eV) and d) N1s (394‐404 eV) regions; e) FTIR spectra of raw materials and PGUA films; f) Typical digital pictures of PGUA‐1 before and after undergoing 72 h in DMF at 60 °C; g) Gel contents of PGUAs in EtOH, PhMe and DMF at RT and 60 °C.

Following successful verification of small molecule, GUAs were synthesized in‐situ through a facile reaction between AG and tri‐HDI (**Figure** **1**a), and were evenly distributed throughout the polymer networks. D230 was used to regulate the cross‐link density and GUAs contents of polymer networks. When the feed molar ratios of AG and D230 are 8:2, 9:1, and 10:0, the proportions of GUAs in networks are 18.7%, 21.5%, and 24.8% (Table S1, Supporting Information). For simplicity, these three cured resins are designated as PGUA‐0.8, PGUA‐0.9, and PGUA‐1. PGUA films of 0.05 to 0.1 mm were perfectly prepared through casting as shown in Figure 1a. The chemical structure and network completion of PGUAs were analyzed using spectroscopic research methods. The formation of GUAs in PGUA films was verified by X‐ray photoelectron spectra (Figure 1b–d). The C1s spectra (Figure 1c) exhibits four main contributions, with the strongest one at 285.0 eV, corresponding to *C‐C* and *C‐H*, and the second at 286.4 eV, originating from *C‐N* in the polymer networks. The remaining two contributions mainly come from the formation of *N = C‐N* and urea bonds in networks accompanied by the addition polymerization reaction of amino and isocyanate, respectively corresponding to 288.5 eV and 289.5 eV.^[^ ^21^ ^]^ The N1s spectra (Figure 1d) further confirmed the formation of GUAs benefitting from its clear recording, with two main contributions at 399.7 eV and 398.5 eV attributed to *N = C‐N* and *C‐N* in PGUAs respectively.^[^ ^22^ ^]^ Moreover, the FTIR spectra (Figure 1e) of AG, tri‐HDI, D230, and PGUAs were collected. The strong band representing stretching vibration of isocyanates displayed in tri‐HDI monomer ranges from 2200 to 2400 cm^−1^, which is absent in the polymer due to the complete reaction of isocyanate groups. The free *N‐H* strong band in AG monomer also completely disappeared in PGUAs at 3440 cm^−1^, and was replaced by the associative *N‐H* absorption wide peak at 3240 cm^−1^.^[^ ^23^ ^]^ The formation of urea bonds promotes the strengthening of the strong band representing the stretching vibration of *C = O* at 1690 cm^−1^ in the polymer, and the variable band representing the stretching vibration of *C = N* at 1630 cm^−1^ is also retained.^[^ ^24^ ^]^ As shown in Figure 1f, PGUA‐1 film was immersed in DMF at 60 °C for 72 h without further dissolution. We then chose the common solvents EtOH, PhMe, and DMF to calculate the gel content of PGUAs (Figure 1g). The gel content of PGUAs in three solvents exceed 85% at 60 °C, and is more than 90% at RT, which indicates that highly crosslink networks are formed. The gel content of PGUAs basically conforms to its cross‐link density law. There were abnormal patterns of PGUA‐0.9 and PGUA‐1 in DMF at room temperature, which may be attribute to the comprehensive influence of temperature, solvent type, and crosslinking density on the soluble substances in networks.

### 2.2 Advanced Physical Properties

**Figure 2:**
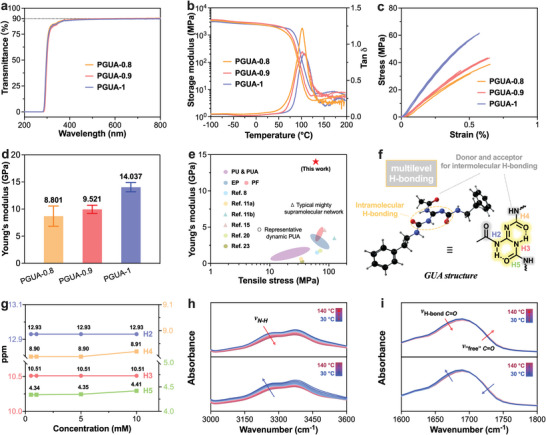
Thermal and physical characteristics of PGUAs. a) UV‐vis transmission spectra, b) DMA curves, c) Stress‐strain curves, and d) Young's modulus histograms of PGUAs; e) Tensile strengths and Young's modulus of PGUAs compared with currently widely used thermosetting plastics and other reported polymers of the same type. f) The ball‐and‐stick models of model compound 2 and structural formulae of GUAs in it, as well as multilevel H‐bond schemata including rigid fused cyclic intramolecular H‐bonding formed by H2‐3 and intermolecular H‐bonding capably formed by H3‐4 with H‐bonding receptors in the network; g) Results of concentration‐dependence of reactive N‐H in small molecule 2; In‐situ FTIR spectra of PGUA‐1 displaying h) 3000–3600 cm^−1^ and i) 1600–1800 cm^−1^ regions.

The PGUAs based on GUAs exhibit several advanced physical properties. The first notable feature is their colorless and transparent nature (Figure 1a). This means that the membrane materials made from PGUAs do not have any inherent color and allow light to pass through them without significant distortion. The UV‐visible transmission spectra of the PGUA membranes shows that they have a high transmittance in visible light ranges, exceeding 90% as indicated in **Figure** **2**a and **Table** **1**. The dynamic mechanical analysis (DMA) curves shown in Figure 2b indicate the behavior of the materials in tensile mode. The different PGUA samples, such as PGUA‐0.8, PGUA‐0.9, and PGUA‐1, exhibit tan *δ* peaks at temperatures of 101, 102, and 108 °C. These peaks correspond to the *T*_g_s of PGUAs as determined by DMA. Figure S4a (Supporting Information) displays the differential scanning calorimetry (DSC) curves, which reveal distinct “steps” at temperatures of 73, 80, and 84 °C. These “steps” also correspond to the *T*_g_s as recorded by DSC. The performance of PGUAs is closely linked to the rigid GUAs and the cross‐link density in the network.^[^ ^25^ ^]^ In Table 1, it is observed that PGUA‐1 outperforms the other groups in terms of thermal properties. A higher proportion of D230 indicates a lower content of GUAs and cross‐link density, a negative correlation with the *T*_g_s. This relationship is reflected in the mechanical properties of the material as well. Figure 2c depicts the stress‐strain curves of PGUAs obtained from a universal testing machine (Figure S5, Supporting Information). The average tensile strength of PGUA‐1 reaches 60 MPa (Table 1). Furthermore, Young's modulus is a significant measure of a material's stiffness or ability to resist deformation under stress, and the maximum modulus of PGUA‐1 exceeds 14 GPa (Figure 2d and Table 1), which is unprecedented among polymers of the same type reported to date (Figure 2e).

**Table 1:** Physical and mechanical properties of all samples.

Samples	*T*_g_ [°C]^a)^		*E*′ [MPa]^b)^	*M*_c_ [g mol^−1^]^c)^	*v*_e_ [mol m^−3^]^d^ ^)^	WCA [°]^e)^	Transmittance [%]	Young's modulus [MPa]	Tensile strength [MPa]	Elongation at break [%]
	DMA	DSC								
PGUA‐0.8	101	73	4.53	428	630	77.6 ± 1.9	91.58	8801 ± 2048	35 ± 3	0.57 ± 0.06
PGUA‐0.9	102	80	5.99	565	643	76.8 ± 2.7	91.38	9521 ± 909	44 ± 7	0.56 ± 0.10
PGUA‐1	108	84	8.03	747	656	76.0 ± 1.0	90.78	14.037 ± 0.850	60 ± 4	0.53 ± 0.05

^a)^ Glass transition temperature;

^b)^ Storage modulus at *T*_g_ + 50 °C;

^c)^ Average molecular weight between cross‐linked points;

^d)^ Cross‐link density;

^e)^ Water contact angle.

The ultra‐high Young's modulus of PGUAs may be attributed to the unique structure of guanidine‐based supramolecular systems, such as the quadruple H‐bonding unit UPy.^[^ ^26^ ^]^ GUAs may exhibit rich diversity and possibility in spatial structure and bonding methods, playing a crucial role in establishing strong intermolecular interactions and creating rigid network structures (Figure 2f). To verify the above inference, model compound 2 with a GUA structure was synthesized through the addition reaction of AG and phenylethyl isocyanate (Scheme S2, Supporting Information). The confirmation of the chemical structure using NMR spectra ensures the accuracy of the following analysis (Figures S2 and S3, Supporting Information). Compound 2 with various concentrations of 10, 5, 1, and 0.5 mm were tested by NMR (Figure S6, Supporting Information). The chemical shifts of H2 (12.93 ppm) and H3 (10.51 ppm) remaining constant at different molecular concentrations indicated the presence of specific intramolecular H‐bonding (Figure 2g).^[^ ^27^ ^]^ Furthermore, according to density functional theory analysis, the intramolecular hydrogen bond energy of rigid repeating unit GUAs is 1.2245 eV (Figure S7, Supporting Information). H3 forms H‐bond with the length of 1.71 Å and the angle of 140° 19', which is slightly larger than H2's 1.81 Å and 139° 50' (Figure S8, Supporting Information). The calculation results also indicated the incomplete symmetry of the spatial arrangement of H2 and H3 H‐bonding. Moreover, H4 and H5 can act as H‐bond donors, while *C = O* in the acetyl group can act as H‐bond acceptors, contributing to the abundant intermolecular H‐bond sites in GUAs. The above analysis of chemical shifts of specific protons (H2, H3, H4, and H5) provides evidence for the formation of specific intermolecular H‐bonding and a H‐bond ring structure in GUAs. Multilevel H‐bond structures enhance the structural stability and stiffness of the material, leading to the exceptionally high modulus observed in PGUAs at the macro level. The in‐situ FTIR spectra (Figure S9, Supporting Information) of PGUA‐1 showed that as the temperature increased, the binding *N‐H* at 3240 cm^−1^ shifted toward higher wavelengths (Figure 2h), and the bonded *C = O* stretching vibration peak (from 1650 to 1700 cm^−1^) decreased while the “free” *C = O* stretching vibration peak (from 1700 to 1750 cm^−1^) increased (Figure 2i).^[^ ^25^ ^]^ These changes indicated that as the temperature increased, the strength of the H‐bond interactions between the oxygen and hydrogen atoms decreased. As a result, the H‐bond networks became disrupted, leading to changes in the vibrational frequencies of the molecular bonds involved. Notably, when the temperature decreased to the initial temperature, the FTIR spectra during the cooling stage exhibited opposite changes to the heating stage (Figure 2h,i), indicating the high reversibility of the multidimensional H‐bond networks in PGUAs.

**Figure 3:**
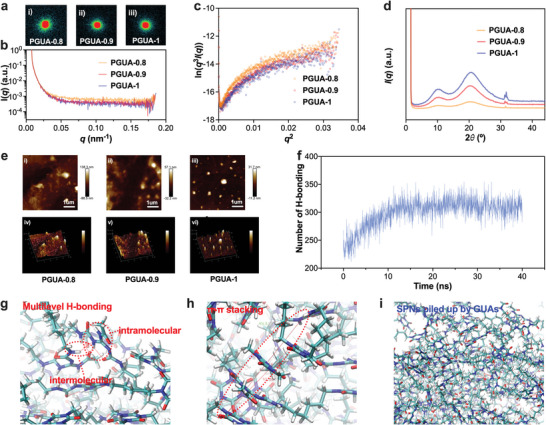
Aggregate structure in PGUAs. 1D a) and 2D b) SAXS profiles of PGUAs; c) Porod fitting curves of PGUAs; d) 1D WDXS profiles of PGUAs; (e) AFM images of PGUAs, including height sensor (i‐iii) and 3D morphology (iv‐vi); f) The number of H‐bonding curves in the MD simulation process; Snapshots of the MD simulations g–i) showing non‐covalent interactions in PGUA networks including intramolecular and intermolecular H‐bonding, and partial π‐π stacking.

Noncovalent interaction aggregates (NIAs) play a significant role in the mechanical properties of polymer materials.^[^ ^28^ ^]^ In the case of H‐bond aggregates or electrostatic interacting aggregates, materials may exhibit some unexpected properties. 1D and 2D small angle X‐ray scattering (SAXS) profiles (**Figure** **3**a,b) of PGUAs were analyzed. 1D scattering curves of PGUAs exhibits a typical small angle scattering behavior, where the intensity (*I*) decreases as the scattering vector (*q*) increases (Figure 3b). This behavior suggests the presence of scatterers with uneven electron density at the nanoscale, which are caused by NIAs.^[^ ^29^ ^]^ 2D scattering patterns reveals that PGUA‐0.8 exhibits a divergent pattern in its outer ring, while PGUA‐1 does not show this feature (Figure 3a). This difference indicates that the scatterer distribution in PGUA‐1 is more uniform, and its structure is more regular than PGUA‐0.8. Therefore, the content of D230 influences the regularity of the NIA structure and its distribution uniformity within materials. Porod curves of PGUAs are derived by processing the 1D scattering intensity curves *I*(*q*) − *q* as *ln*(*I*(*q*)*q* ^3^) − *q* ^2^.^[^ ^30^ ^]^ In the high‐angle region, these curves appear as a straight line with a positive slope (Figure 3c), indicating positive deviation. This suggests that the scatterer and the matrix behave as a nonideal two‐phase system.^[^ ^29b^ ^]^ Besides NIAs, there may be additional factors contributing to the material structure, such as uneven electron density or thermal density fluctuation. The sharp peaks observed at 32° in wide angle X‐ray scattering (WDXS) profiles provide further evidence of the existence of NIAs in the material structure (Figure 3d; Figure S11, Supporting Information),^[^ ^15a^ ^]^ with GUAs playing a significant role. To directly confirm the nanoscale NIAs in PGUAs, atomic force microscopy (AFM) was used to observe casting micron order films on a glass plate. The height sensor (Figure 3e‐i‐iii) and 3D morphology (Figure 3e‐iv‐vi) images revealed that the scatterers in the material structure of PGUA‐1 were more uniformly distributed and displayed distinct morphological features compared to the other two groups. These scatterers appeared as white spots ranging in size from tens to hundreds of nanometers. Moreover, PGUA‐0.8 and PGUA‐0.9 exhibited local aggregation of continuous scatterers with strong inhomogeneity. This means that these two groups showed clusters or regions where the scatterers were densely packed and exhibited a lack of uniform distribution. These observations are consistent with the previous findings mentioned, suggesting that PGUA‐1 has a more regular and uniform structure compared to PGUA‐0.8 and PGUA‐0.9. Molecular dynamics (MD) simulations were utilized to assist in analysis the weak interactions of PGUAs. In addition to multilevel H‐bonding, the structural unit GUAs in networks occurred π‐π stacking due to its own aromaticity. The polymer network was created through the self‐assembly of 10 supramolecular polymer units within a 12 nm cube, utilizing PGUA‐1 as the building blocks (Figure S12, Supporting Information). The Materials Studio software was employed to detect the number of H‐bonding within the network simultaneously. During the network assembly process, the number of H‐bonging increased from 223 to 332 (Figure 3f). Among these, 223 corresponded to intramolecular H‐bonding within GUAs, while the increment was associated with the formation of intermolecular H‐bonding. These are in agreement with the results obtained by the model compound experiments and in‐situ FTIR spectra (Figure S9, Supporting Information). Snapshots of the MD simulations are provided in Figure 3g–i and Figure S13 (Supporting Information). In these figures, short red rods represent O, white rods represent H, blue three‐branch rods represent N, and green four‐branch rods represent C. Figure 3g demonstrates the formation of the multilevel H‐bond network consisting of both intramolecular and intermolecular H‐bonding, as marked by the red dotted lines. In Figure 3h and Figure S13 (Supporting Information), the face‐to‐face and edge‐to‐face π‐π stacking of GUAs is depicted as the red circles. This stacking occurs when aromatic moieties align and interact through π electron interactions. The two dispersion peaks in Figure 3d correspond to the weak interaction in the network. Calculated according to Bragg's law the scattering vector *q* = (4π/λ)sin2θ, where *λ* is 1.54 Å, 2*θ* is the scattering angle. The maximum scattering vector value was 0.5 Å^−1^. Among them, 0.6993 nm corresponds to the double stacking of 1, 3‐diamino‐5‐triazine rings in the network, while 1.4286 nm comes from GUAs and its multiple stacking (Figure S13, Supporting Information). The π‐π stacking also is an important driving force for the formation and stabilization of PGUAs network. The formation of the PGUAs network is a result of the combined action of multilevel H‐bonding and π‐π stacking (Figure 3i). The π‐π stacking provide additional stability to the network by forming local directional bonds between the unit GUAs. These weak interactions contribute to local clustering and scattering of the scatterers within the network, leading to the overall superior and especially ultra‐modulus properties observed.

### 2.3 Dynamic Nature

**Figure 4:**
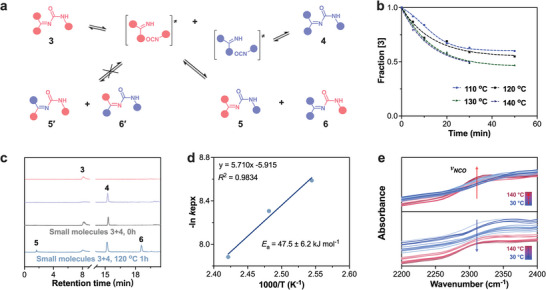
Dynamic exchange reaction of GUAs models. a) Schematic diagram of the exchange reaction between small molecules 3 and 4; b) Exchange kinetics between small molecules 3 and 4 at different temperatures; c) LC‐MS spectra of small molecules 3, 4 and 3+4 before and after the reaction; d) Arrhenius plot of 3 and 4 exchange kinetics and activation energy (*E*_a_); e) In‐situ FTIR spectra of PGUA‐1 showing 2200–2400 cm^−1^ regions.

Guanamine urea bond in GUAs, which are formed by the reaction of *C = NH* with an isocyanate group, are indeed considered to belong to the category of dynamic urea chemistry due to formation mechanism and its chemical structure like pyrazole‐urea structures.^[^ ^11b^ ^]^ Therefore, we focused on its dynamics of exchange response. Model compounds 3 and 4 (Schemes S3 and S4, Supporting Information) containing guanamine urea bonds were synthesized, and their chemical structures were determined by NMR spectra (Figures S14–S17, Supporting Information). Combining them in a 1:1 stoichiometric ratio, the double replacement reaction of the guanamine urea bonds was able to be confirmed (**Figure** **4**a). To analyze the exchange reaction, we conducted experiments at various temperatures ranging from 110 to 140 °C (Figure 4b). LC‐MS analysis was used to monitor the reaction, focusing on the characteristic signals of the original small molecules 3 and 4 (Figure 4c; Figure S18, Supporting Information). As the temperature increased and the reaction time prolonged, a decrease in the intensity of the above two signals was observed, indicating the consumption of small molecules 3 and 4. Furthermore, the signal peaks of the exchange reaction products, small molecules 5 and 6, were detected (Figure 4c; Figure S18, Supporting Information). These signals gradually increased with higher temperatures and longer reaction times, suggesting the formation of the exchange products. As envisaged, it is found that the signals corresponding to small molecules 5' and 6' were extremely weak (Figures S18 and S19, Supporting Information), indicating that the metathesis reaction of the guanamine urea bonds in GUAs was most significant under temperatures below 140 °C. Immediately after, that have fitted the kinetic data of the guanamine urea bond exchange reaction and obtained the reaction rate constant *k* at various temperatures (Figure S20, Supporting Information). By plotting these rate constants in an Arrhenius plot, it computed that the activation energy for the exchange reaction is 47.5 ± 6.2 kJ mol^−1^ (Figure 4d). These conjugate structures in GUAs can affect the dynamic behavior of urea, making them more likely to undergo exchange reactions.

During the heating and cooling phases, in‐situ FTIR spectra (Figure 4e) on PGUAs are indeed indicative of the guanamine urea bond dissociation exchange reaction. The significant broadband intensification of isocyanate stretching vibrations in the 2200 to 2400 cm^−1^ region during the heating phase suggests the formation of new isocyanate species as a result of the dissociation of guanamine urea bonds (Figure 4e).^[^ ^25^ ^]^ This broadband intensification indicates an increase in the concentration of isocyanate groups, which are characteristic of the dissociated guanamine urea bonds. Moreover, during the cooling stage, the observed weakening and disappearance of the broadband suggests the reverse process. It indicates the slower and slower dissociation of the guanamine urea bonds, leading to the decrease or absence of concentration of isocyanate. The high degree of reversibility observed in this process supports the conclusion that the broadband intensification and subsequent weakening even disappearance are due to the dissociation and reformation of guanamine urea bonds highly depending on temperature. The rheological behavior of PGUAs involved two fast drops in storage modulus (Figure S21, Supporting Information). The first drop occurred at 50 °C, which corresponds to the glass transition of the polymers. This transition occurs when the polymer chains transition from a “frozen” state to a mobile state, leading to a rapid decrease in modulus.^[^ ^31^ ^]^ The second rapid drop in modulus occurred at 170 °C. This corresponds to the topological freezing transition, which occurs when the crosslinking network collapses due to the dissociation of guanamine urea bonds. This collapse leads to a rapid decrease in modulus, as the polymer material loses much of its network structural integrity.^[^ ^32^ ^]^

**Figure 5:**
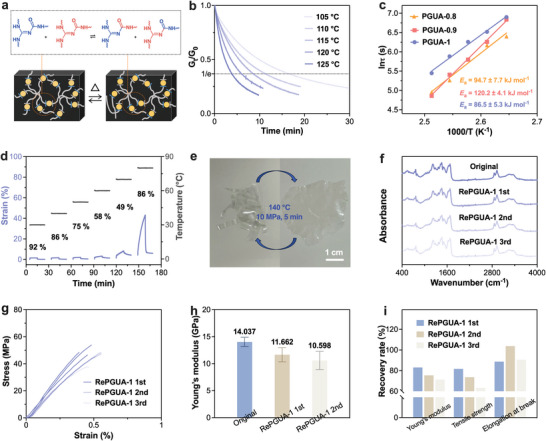
Dynamic nature of PGUAs. a) Diagram of guanamine urea bonds metathesis and PGUA networks rearrangement; b) Stress relaxation curves of PGUA‐1 at varying temperatures; c) *ln*τ as a function of 1000/*T* for all samples based on Arrhenius; d) TTS creep curves for PGUA‐1; e) Typical photos of reprocessing PGUA‐1; f) FTIR spectra of the reprocessed PGUA‐1; g) Stress‐strain curves of the reprocessed PGUA‐1; h) Relative Young's modulus histograms of PGUA‐1 and different reprocessing circles PGUA‐1; i) Mechanical properties recovery rate of PGUA‐1 at different reprocessing circles.

Under certain conditions that affect the guanamine urea bonds metathesis (**Figure** **5**a), such as high temperature, a high degree of dissociation can lead to the collapse of the crosslinking network, resulting in a transition from a gel‐like state to a sol‐like state.^[^ ^33^ ^]^ However, within the temperature range studied, the Arrhenius behavior can still be observed. This is because although there is sufficient distribution between open and closed cross‐links to allow for rapid exchange, it is negligible in terms of changing the network structure and overall crosslinking density.^[^ ^34^ ^]^ In Figure 5b and Figure S22 (Supporting Information), stress relaxation experiments on PGUAs were conducted using DMA subjected to a 3% strain at varying temperatures. The stress of all samples exhibited an exponential decrease with time. It can be described by the Maxwell model with a single characteristic relaxation time (τ*), which is defined as the time required for the stress relaxation curves to reach 1/e of its original stress value.^[^ ^35^ ^]^ All PGUAs showed significant stress relaxation, and the relaxation occurred faster with increasing temperature. In the case of PGUA‐1, as the temperature increased from 105 to 125 °C, the τ* decreased from 16.6 to 3.9 min (Figure 5b). The rate of stress relaxation is influenced by the mobility of the chemical species involved in exchange reactions.^[^ ^36^ ^]^ At the same temperature, the stress relaxation rates of PGUA‐0.8 and PGUA‐0.9 were faster compared to PGUA‐1 (Figure S22, Supporting Information). This could be attributed to the addition of polyether amine, which appropriately reduces the network cross‐link density. This increased chain‐segment mobility for PGUA‐0.8 and PGUA‐0.9 compared to PGUA‐1. However, excessive polyether amine can decrease the distribution of guanamine urea bonds within the networks, which may explain why the relaxation rate of PGUA‐0.9 is faster than that of PGUA‐0.8 at 125 °C. In the case of PGUA samples with different compositions, such as PGUA‐1, PGUA‐0.9, and PGUA‐0.8, the activation energies (*E*_a_) for bond exchange reactions were calculated according to Arrhenius.^[^ ^37^ ^]^ The results show that PGUA‐1 has the lowest *E*_a_ (86.5 ± 5.3 kJ mol^−1^) (Figure 5c), indicating a higher activity of the bond exchange reaction in the network compared to the other samples. When D230 is participated in building to the PGUA networks, the concentration of guanamine urea bonds decreases and ordinary urea bonds increases. This leads to a higher *E*_a_ for PGUA‐0.9 (120.2 ± 4.1 kJ mol^−1^) compared to PGUA‐1. For PGUA‐0.8, which has a lower cross‐link density compared to PGUA‐0.9, the *E*_a_ decreases to 94.7 ± 7.7 kJ mol^−1^ (Figure 5c). This suggests that the further decrease in cross‐link density increases the probability of guanamine urea bonds exchange. In summary, the differences in *E*_a_s among different components may be due to chain‐segment mobility otherness and guanamine urea bonds of local concentration differences.

DCPs are generally known to have poorer dimensional stability compared to traditional thermosets.^[^ ^38^ ^]^ Meanwhile, Figure 5d and Figure S23 (Supporting Information) show the time‐temperature‐superposition (TTS) creep curves of PGUAs. The figures show that PGUA‐0.8 and PGUA‐0.9 have better creep resistance because of deformation recovery of over 95% below 50 °C. However, PGUA‐1 exhibits a better deformation recovery rate of 86% at 80 °C. This pattern conforms to the proportion rule of GUAs containing guanamine urea bonds, indicating that the differences in deformation behavior can also be attributed to the differences in the composition and chain‐segment property of the PGUAs. The dynamic nature of guanamine urea bonds in GUAs allows the network topology to be rearranged while maintaining its cross‐linked structure (Figure 5a), enabling the PGUAs to be reprocessed effectively. In the embodiment, as shown in Figure 5e, PGUA‐1 fragments were hot pressed for 5 min at 140 °C and 10 MPa to obtain a complete film. In the FTIR spectra (Figure 5f), there was no significant difference in the chemical structure of the samples. However, some changes in mechanical properties were observed, such as a slight increase in modulus of PGUA‐0.8 and PGUA‐0.9 after the first reprocessing (Figure S24 and Table S2, Supporting Information), which could be attributed to a minor hardening of the plastic during hot pressing. After three hot‐pressed treatments, the mechanical properties of PGUAs showed a significant loss due to serious thermal degradation (Figure 5g; Figure S24, Supporting Information). The Young's modulus, tensile strength, and elongation at break decreased, but there were still certain levels of recovery observed and maintaining a high modulus exceeding 10 GPa (Figure 5h; Table S2, Supporting Information) especially PGUA‐1. The recovery rates for Young's modulus, tensile strength, and elongation at break were 71.4%, 63.3%, and 90.6% respectively (Figure 5i; Table S2, Supporting Information).

### 2.4 Antibacterial Characteristic

**Figure 6:**
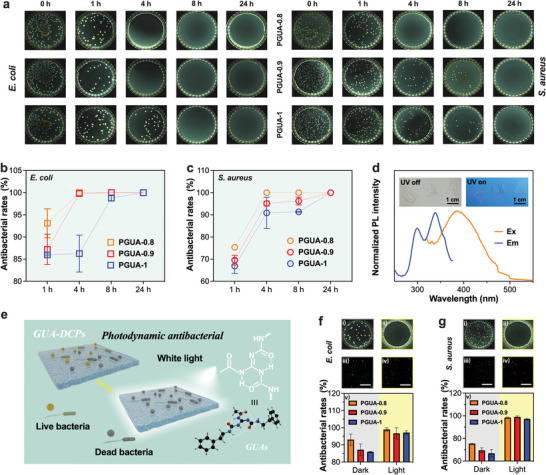
Contact and AIE‐active antibacterial. The digital photos a) and antibacterial rate b,c) of PGUAs on *E. coil* and *S. aureus* at 0 h, 1 h, 4 h, 8 h, and 24 h by the spread plate method; d) Fluorescence emission and excitation spectra of PGUA‐1, and its photographs under the 365 nm UV lamp on and off environments; Schematic diagram e) of contact and AIE‐active antimicrobial concurrent on PGUA membranes with GUAs; The spread plate pictures (i‐ii) and live/dead double staining images (iii‐iv) on *E. coli* f) and *S. aureus* g) under dark and light conditions of 1h each after treated with coated PGUA‐1 films, and position the relative antibacterial activity bar chart (v) below them. The unmarked scale bars represent 500 µm.

Guanidine‐based chemicals or polymers have indeed garnered significant attention as a new generation of biocides. In addition to their efficacy against drug‐resistant bacteria, guanidine‐based chemicals and polymers also possess favorable attributes such as good biocompatibility, low toxicity, and excellent antibacterial activity.^[^ ^39^ ^]^ Nevertheless, it is still used as an organic antibacterial agent and has never been reported for the direct preparation of antibacterial plastics. We investigated the antibacterial characteristic of PGUAs. *E. coil* and *S. aureus* were selected as typical representatives of Gram‐negative and Gram‐positive bacteria to assess the antibacterial effects of the PGUA membranes in vitro and their potential as antimicrobial materials. The antibacterial effect and time correlation of PGUA membranes were studied (**Figure** **6**a; Figure S26, Supporting Information), and it was found that over time, the antibacterial effect of all samples became stronger. The antibacterial rate under different conditions was calculated using the coating time of 0 h as a control. The antibacterial rates of PGUA‐0.8, PGUA‐0.9, and PGUA‐1 against *E. coli* were 93.1 ± 3.3%, 87.2 ± 3.4%, and 85.9 ± 0.2% (Figure 6b), respectively after 1 h. After 4 h, PGUA‐0.8 reached 100%, while after experiencing 8 h, PGUA‐0.9 also reached 100% (Figure 6b). At these two time points, the antibacterial rates of PGUA‐1 were 86.3 ± 4.2% and 98.8 ± 0.7% (Figure 6b). All samples have reached 100% after 24 h (Figure 6b). In addition, consistent antimicrobial effects of PGUA membranes against *S. aureus* were observed (Figure 6c). The concentration of GUAs and the amount of D230 in the membrane were closely related to the antibacterial activity. PGUA‐0.8 had the best antibacterial effect due to the greater contribution of polyether amines to the antibacterial effect, while the antibacterial effect of PGUA‐1 was entirely attributable to GUAs. The above results verify that PGUAs have good antibacterial properties and showed significant inhibition against *E. coli* and *S. aureus*.

One specific type of luminescent material is known as cluster‐induced luminescence (CTE) materials having gained significant attention from researchers in recent years. Unlike traditional materials that rely on large, conjugated structures, CTE materials consist of aggregated electron‐rich chromophore clusters. These chromophores undergo intermolecular and intramolecular interactions, resulting in group clustering and conformational hardening.^[^ ^40^ ^]^ Ultimately, this makes them susceptible to UV light excitation and the subsequent emission of fluorescence. The UV absorption peaks at 231 and 286 nm suggest that PGUAs absorb UV light at those specific wavelengths (Figure S27, Supporting Information). Under 365 nm UV light irradiation, PGUAs exhibit photoluminescence and emit blue fluorescence (Figure 6d). The fluorescence spectrum shows that PGUAs have optimal excitation and emission wavelengths at 343 and 392 nm (Figure 6d; Figure S28, Supporting Information). This fluorescence emission is a result of the absorbed energy being released as light, which may be attributed to the GUA structure. The interaction of multilevel H‐bonding and the π‐π stacking from GUAs likely contribute to the aggregation of chromophores and conformational hardening (Figure 6e).^[^ ^41^ ^]^ This limits molecular motion, suppresses non radiative transitions, and simultaneously promotes radiative transitions under specific wavelength.^[^ ^42^ ^]^ At this point, the high concentration of charges on the surface of PGUA membranes can significantly enhance the electrostatic interaction with bacterial cytomembrane and tear it apart more efficiently (Figure S29, Supporting Information),^[^ ^43^ ^]^ while being irradiated by stimulated radiation transitions, achieving more effective photodynamic sterilization (Figure 6e).^[^ ^44^ ^]^ In the repeated experiment conducted for 1 h in a white light incubator, we conducted plate coating and live/dead double staining (Figure 6f–i–iv, Figure 6g–i–iv, and Figure S30, Supporting Information). It was observed that the antibacterial rates of all samples were improved, while there was no effect on bacterial activity from a white light (Figure S31, Supporting Information). Specifically, for *E. coli*, the antibacterial rates of PGUA‐0.8, PGUA‐0.9, and PGUA‐1 increased from 93.1 ± 3.3%, 87.2 ± 3.4%, and 85.9 ± 0.2% to 99 ± 1.0%, 96.7 ± 3.3%, and 97.2 ± 1.0% (Figure 6f–v). Similarly, for *S. aureus*, the antibacterial rates of PGUA‐0.8, PGUA‐0.9, and PGUA‐1 increased from 75.3 ± 0.2%, 69.6 ± 2.2%, and 66.9 ± 3.4% to 98.5 ± 0.5%, 99 ± 0.9%, and 97.1 ± 0.4% (Figure 6g–v). The increased antibacterial rates indicate a more effective destruction of bacteria, both for *E. coli* and *S. aureus*.

Reactive oxygen species (ROS), such as singlet oxygen and hydroxyl radicals, are the key mediators in photodynamic antibacterial that cause damage to bacterial cells, leading to their destruction. The comparison of the light treatment group and the dark control group is essential for determining whether the observed fluorescence is due to ROS production induced by light exposure. The large areas of green fluorescence in the light treatment group suggest significant ROS generation (Figure S32, Supporting Information), highlighting the effectiveness of the photodynamic process in producing ROS. Furthermore, the observation that samples with a higher ratio of GUAs produced more ROS indicates the influence of the photosensitizer content on ROS generation. The 3D image in Figure S33 (Supporting Information) offers a visual representation of the PGUAs biofilms. It is notable that PGUAs can effectively reduce the presence of E. coli or S. aureus within biofilms upon irradiation with white light for 1 h. This suggests that ROS play a significant role in inducing bacterial death within biofilms when exposed to the white light. This photodynamic antibacterial is more conducive to the widespread use of PGUAs in daily life, especially when exposed to natural light. By curing PGUA‐1 onto the surface of a keyboard key and allowing it to be used for two weeks, then its antibacterial activity was assessed (Figure S34, Supporting Information). The observed excellent antibacterial properties of the treated keyboard key suggest that PGUAs have the potential as antibacterial plastics. Notably, it is that the inhibitory effect of PGUAs on bacteria was not significantly lost after reprocessing (Figure S35a, Supporting Information). The inhibition rate against *E. coli* remained at 100% after 24 h even after two reprocessing treatments (Figure S35b, Supporting Information). After the third reprocessing, the bacteriostatic rate of all the samples remained above 99% (Figure S35b, Supporting Information). These results elucidate the nice recycling and reusing abilities of PGUAs.

## 3 Conclusion

In summary, GUAs, a novel dynamic covalent supramolecular structure that offers a unique combination of high performance and sustainability, were formed through a simple addition polymerization reaction between AG and tri‐HDI. The ratio of GUAs in the network can be adjusted by using D230, which influences both the microstructure and macroscopic properties of GUA‐SPs. Results by various characterization techniques, such as in‐situ FTIR spectra, MD simulations, SAXS and WDXS, and AFM have confirmed the formation of NIAs driven by multidimensional H‐bonding and strengthened by π‐π stacking interactions. As a result, the material showed an extremely high Young's modulus of 14 GPa. Additionally, the materials exhibited reconfiguration properties after mechanical crushing. Model compounds, and the temperature dependent infrared and rheological behavior of GUA‐DCPs were used to demonstrate the dynamic separation and association ability of guanamine urea bonds in GUAs, which was proven to follow the dissociative exchange mechanism. Furthermore, GUAs possessed universal antibacterial properties due to the presence of imine bond. The antibacterial properties of GUA‐SPs have been thoroughly verified, and it is noteworthy that the weak interaction clusters within GUAs enabled photodynamic antibacterial capabilities. GUAs have enormous potential in the fields of energy, environment, and healthcare, and their dynamic characteristics, adjustable structures, and unique performance combinations make them promising candidates for various technological advancements.

## Conflict of Interest

The authors declare no conflict of interest.

## Supporting information

Supporting Information

## Data Availability

The data that support the findings of this study are available from the corresponding author upon reasonable request.
