# Supplementary material for: Malleable, Ultrastrong Antibacterial Thermosets Enabled by Guanidine Urea Structure
Source: Adv Sci (Weinh). 2024 Jun 13;11(30):2402891. doi: 10.1002/advs.202402891 (PMC11321644; doi:10.1002/advs.202402891)
Supplement: Supplementary file 1 — Supporting Information [file ADVS-11-2402891-s001.docx]

***Supporting information***

**Malleable, ultrastrong** **antibacterial thermosets enabled by** **guanidine urea structure**

*Zhen Yu,^1^ Qiong Li,^1^ Yanlin Liu,* Shu Tian, Wanding Chen, Yingying Han, Zhaobin Tang* and Junping Zhang**

1. **Supplementary Methods**

**Materials.** All the raw materials, including *N*-phenylurea (Aladdin, 97%), benzaldehyde (Aladdin, 99.5%), 1,1,3,3-tetramethylguanidine (Aladdin, 99%), 1,3-diphenylguanidine (Aladdin, 97%), ethyl isocyanate (Aladdin, 99%), phenethyl isocyanate (Aladdin, >98%), amino-terminated polyoxypropylene (D230, Aladdin, Mn ≈ 230 g mol^-1^), acetonitrile (Aladdin, standard for GC), 1-acetylguanidine (AG, Macklin, 98%), hexamethylene diisocyanate trimer (tri-HDI, Macklin), methanol (Sinopharm, AR), ethanol (EtOH, Sinopharm, AR), toluene (PhMe, Sinopharm, AR), N, N’-dimethylformamide (DMF, Sinopharm, AR), petroleum ether (Sinopharm, 60~90 °C), dichloromethane (DCM, Sinopharm, AR) and trichloromethane (CHCl_3_, Sinopharm, AR) were used without further purification.

**Synthesis of 1,1'-(phenylmethylene)bis(3-phenylurea)^[1]^ (Small molecule 1).** 1.36 g (0.01 mol) *N*-phenylurea, 1.06 g (0.01 mol) benzaldehyde and 20 mL methanol were placed in a 50mL round-bottomed flask and refluxed at 50 °C for 6 h. After the fulfillment of reaction via notice of TLC, 1.79 g white solid was separated out, which was filtered, dried, and recrystallized in CH_3_OH/CHCl_3_ (v/v, 1:1) to yield of 74% pure small molecule 1. The resulting route is shown in **Scheme S1**.

^1^H NMR (400 MHz, DMSO-*d*_6_, ppm): δ 8.67 (s, 1H), 7.47–7.40 (m, 2H), 7.34–7.21 (m, 3H), 7.09 (d, *J*=8.0 Hz, 1H), 6.95–6.87 (m, 1H), 6.35 (t, *J*=8.0 Hz, 1H).

**Scheme S1. S**ynthetic route of small molecule1.

**Synthesis of (*Z*)-*N*-(*N*,*N*'-bis(phenethylcarbamoyl)carbamimidoyl)acetamide (Small molecule 2).** 1.01 g (0.01 mol) AG, 2.94 g (0.02 mol) phenyl ethyl isocyanate and 50 mL DMF were added into 100 mL round-bottomed flask, mixed and stirred for 2h in N_2_ atmosphere. After the reaction, the crude product was poured into an appropriate amount of DCM/petroleum ether (v/v, 1/9) and stirred for 1h. Finally, through liquid phase separation, and then through the rotary evaporator to remove the residual solvent, 3.24 g milky white liquid yield of 82%. The resulting route is shown in **Scheme S2**.

^1^H NMR (400 MHz, CDCl_3_, ppm): δ 12.93 (s, 1H), 10.51 (s, 1H), 8.93 (t, J=5.7 Hz, 1H), 7.36 (q, J=7.9 Hz, 2H), 7.23–7.07 (m, 9H), 4.48 (t, J=6.4 Hz, 1H), 3.70 (q, J=6.0 Hz, 2H), 3.30 (q, J=6.9 Hz, 2H), 2.90–2.68 (m, 5H), 2.18 (s, 3H).

^13^C NMR (101 MHz, CDCl_3_, ppm): δ 171.30, 162.27, 152.28, 151.50, 139.37, 138.90, 128.94, 128.83 (d, J=2.9 Hz), 128.74–128.61 (m), 128.57 (d, J=4.6 Hz), 126.61, 126.25, 41.63, 40.04, 35.94, 35.32, 25.10.

**Scheme S2. S**ynthetic route of small molecule 2.

**Synthesis of 1-(bis(dimethylamino)methylene)-3-phenethylurea (Small molecule 3).** 5.57 g (0.05 mol) 1,1,3,3-tetramethylguanidine and 20 mL DCM were added to a 100 mL three-mouth flask. 7.36 g (0.05 mol) phenethyl isocyanate and 20 mL DCM were put into the dropping funnel, added drop by drop to the system at 5 °C within 30 min, and mixed and stirred for 2h after returning to room temperature. The above process is carried out in N_2_ atmosphere. After the reaction, the crude product was poured into an appropriate amount of petroleum ether and stirred for 1 h. Finally, 9.56 g of straw yellow liquid yield of 74% is through the separation of liquid phase and then remove residual solvent via rotary evaporator. The resultant route is shown in **Scheme S3 (a).**

^1^H NMR (400 MHz, CDCl_3_, ppm): δ 7.31–7.13 (m, 1H), 3.49 (q, *J*=6.8 Hz, 0H), 2.81 (s, 2H).

^13^C NMR (101 MHz, CDCl_3_, ppm): δ 165.03, 162.68, 139.68, 128.85, 128.39, 126.09, 41.72 (d, *J*=12.5 Hz), 39.45, 36.65.

**Synthesis of 1-(bis(phenylamino)methylene)-3-ethylurea (Small molecule 4).** 10.56 g (0.05 mol) of 1,3-diphenylguanidine and 20 mL DCM were added to a 100 mL three-mouth flask. 3.55 g (0.05 mol) ethyl isocyanate and 20 mL DCM were put into the dropping funnel, added drop by drop to the system at 5 °C within 30 min, and then mixed and stirred for 2 h after returning to room temperature. The above process is carried out in N_2_ atmosphere. After the reaction, the crude product was poured into an appropriate amount of petroleum ether and stirred for 1h. Finally, 6.01 g white solid yield of 43% was obtained by filtration and drying by vacuum oven. The resulting route is shown in **Scheme S3 (b)**.

^1^H NMR (400 MHz, CDCl_3_, ppm): δ 9.28 (s, 1H), 7.53–7.26 (m, 6H), 7.11–6.94 (m, 3H), 4.57 (s, 2H), 3.40–3.26 (m, 4H), 1.19 (t, *J*=7.2 Hz, 7H).

^13^C NMR (101 MHz, CDCl_3_, ppm): δ 155.88, 150.04, 138.41, 129.98, 129.73, 129.61, 128.70, 123.20, 122.72, 34.80, 15.08.

**Scheme S3.** Synthetic routes of small molecule 3 and small molecule 4.

**Preparation of PGUAs.** 0.15 g (0.0015 mol) AG was added to 20 mL DMF and stirred until dissolved. 0.529 g (0.00105 mol) tri-HDI was added and continued to stir until evenly. The film was cast at 100 °C, when the solvent is completely volatilized and then cured in a vacuum oven at 120 °C for 2 h. Eventually, the colorless transparent film PGUA-1 was obtained. The preparation process of PGUA-0.9 and PGUA-0.8 requires D230 to be diluted with DMF and next the same as above process. The formulas of PUGAs are shown in **Table S1**.

**Gel content test.** 20 mg of PGUAs was soaked in 10 mL vials filled with EtOH, PhMe and DMF at room temperature and 60 °C for 72 h, and then dried in a vacuum oven at 80 °C for 24 h. The gel content was calculated by $\frac{m_{1}}{m_{0}}\times100\%$. *m*_0_ is the initial mass, and *m*_1_ is the final mass.

**Exchange experiment of GUA model.** 100 mg (0.38 mmol) model compound 3 and 107 mg (0.38 mmol) model compound 4 were weighed and dissolved in a 10 mL vial with 100 uL acetonitrile/deionized water (v/v, 1/1). The above mixture was heated in an oil bath at different temperatures for different times, and then 5 uL of the mixture was diluted with 995 uL acetonitrile/deionized water (v/v, 1/1) for liquid chromatography mass spectrometry detection.

**Dynamic thermal mechanical analysis test.** DMA was performed on Q850 DMA (TA, USA) in a tension mode with dimensions of around 20 mm (length) × 5 mm (width) × 0.1 mm (thickness) to measure the dynamic mechanical properties of PGUAs from -100 to 200 °C with a heating rate of 3 °C min^−1^ at a frequency of 1 Hz and constant amplitude of 20 μm. The actual cross-link density (*v*_e_) of the sample was calculated based on the DMA data through **equation S1**. The average molecular weight between the cross-linked points (*M*_c_) was calculated by **equation S2**, and the theoretical cross-link density of the samples was estimated.^[2]^

$E^{'}=3v_{e}RT$ (**S1**)

$M_{c}=({n_{AG}M_{AG}+n_{D230}M_{D230}+n_{NCO}M_{NCO}}/{n_{NCO}})$ (**S2**)

where *E*′ is the platform storage modulus of the sample at *T* + 50 °C, *R* is the gas constant, and *T* is the absolute temperature. *M* and *n* represent the molar mass and molar concentration of the corresponding components, respectively.

**Antibacterial experiment.** The tests for the antibacterial activity of PGUAs used the spread plate method. The samples were cultured with a concentration of 10^5^ Escherichia coli (*E. coli*) and Staphylococcus aureus (*S. aureus*) suspension for different time periods (0, 1, 4, 8, and 24 h) at 37 °C. The 1 h experimental groups, with consistent repeated conditions, needed to be cultured in white light (6000 Lux light intensity). To prepare for the colony tests, solidified agar plates were created and a 10 µL suspension was spread uniformly across the surface using glass beads. The solidified agar plates containing either *E. coli* or *S. aureus* were then cultured at 37 °C for 24 h. Colony tests were conducted at least two times independently, and the number of colonies was counted using a Scan 300 automatic colony counter (Interscience, France). The antibacterial rate (AR) was computed by the following **equation S3.**

$AR\left( \% \right)={N_{t}}/{N_{0}}$ (**S3**)

where *N*_0_ is the mean value of the colony number at 0 h and *N*_t_ is that at t h. To observe bacterial morphology, all samples were first fixed in 2.5% glutaraldehyde in a refrigerator for 3 h. After the fixed sample was removed, it was washed twice with 0.9% physiological saline. The sample was then dehydrated by immersing it in a series of increasing concentrations of ethanol (25%, 50%, 75%, 90%, and 100%) to facilitate preservation of bacteria structures. The sample was placed in a vacuum oven at 36 °C overnight. Finally, it was coated with a layer of platinum (Pt). The morphology of *E. coli* and *S. aureus* was observed by G4 UC scanning electron microscopy (SEM, Verios, USA) operating at an accelerating voltage of 2.00 kV.

**Reprocessing of PGUAs.** Fragmentary PGUAs were subjected to hot-pressing at 140 °C and 10 MPa for 5 min to obtain the reprocessed PGUAs.

**The test of ROS production.** The concentration of 10^5^ CFU mL^-1^ *E. coli* and *S. aureus* with PGUAs membranes (about 5 mm x 5 mm, length x width) was added to the 96-well plate and incubated at 37 °C for 1 h under light or dark conditions. Then, the samples were gently washed with PBS buffer to remove the floating bacteria on the surface, then incubated with 100 μL 10 μM 2′,7′-dichlorofluorescein diacetate (DCFH-DA, Macklin, 99%) solution at 37 °C for 30 min in a dark environment, and then washed with PBS to remove the residual reagent. The prepared samples were imaged by a fluorescence inverted microscope.

**The antibiofilm test of PGUAs.** The concentration of 10^5^ CFU mL^-1^ *E. coli* and *S. aureus* with PGUAs membranes (about 5 mm x 5 mm, length x width) was added to the 96-well plate and incubated at 37 °C for 1 h under light or dark conditions. The samples were gently washed with PBS buffer to remove the floating bacteria on the surface, then incubated with 100 μL SYTO^TM^ 9 solution (*V*_dye_/*V*_normal saline_,1.5 uL/1 mL) at 37 °C for 30 min in dark, and then washed with PBS to remove the residual reagent. The prepared samples were 3D imaged by a TCS SP5 biotype laser confocal microscope (Leica, Germany).

**Characterizations.** ^1^H NMR and ^13^C spectra were measured via an AVANCE III 400 MHz NMR spectrometer (Bruker, Switzerland) with CDCl_3_ and DMSO-*d*_6_ as solvent. XPS spectra of samples were recorded by an AXIS SUPRA X-ray photoelectron spectrometer (Kratos, UK) with a radiation resource C-O (α). Water contact Angle of films (WAC) was measured with an OCA25 contact angle tester (Dataphysics, Germany). Tensile tests were examined with a Z1.0 universal testing machine (Zwick, Germany). Thermogravimetric experiments and DSC curves of samples were recorded using TGA/DSC1 thermal gravimetric analyzer and differential scanning calorimeter (METTLER TOLEDO, China). Liquid and powder samples were carried out on a NICOLET 6700 intelligent Fourier infrared spectroscopy (THermo, USA), while films were carried out using a Cary660+620 micro-FTIR (agilent, USA). Stress relaxation and TTS creep experiments were recorded with a DMAQ850 dynamic mechanical analyzer (TA, USA). Rheological property of samples was recorded using a HR-3 rotational rheometer (TA, USA). The transmittance of samples was measured by a Lambda 950 UV-VIS-NIR Spectrophotometer (Perkin-Elmer, USA). Liquid chromatography mass spectrometry (LC-MS) spectra were detected by a LC-MS analyzer (AB Sciex, USA). SAXS and WDXS profiles were recorded with a Xeuss 3.0 UHR 2D small angle X-ray scatterometer (XENOCS SAS, France) with incident wavelength $\lambda=1.54 Å$. AFM morphology images were recorded using a Dimension ICON Scanning probe microscope (Bruker, USA). Live/dead double staining images on bacteria were recorded with Ti2-U inverted fluorescence microscope (Nikon, Japan).

1. **Supplementary Figures**

**
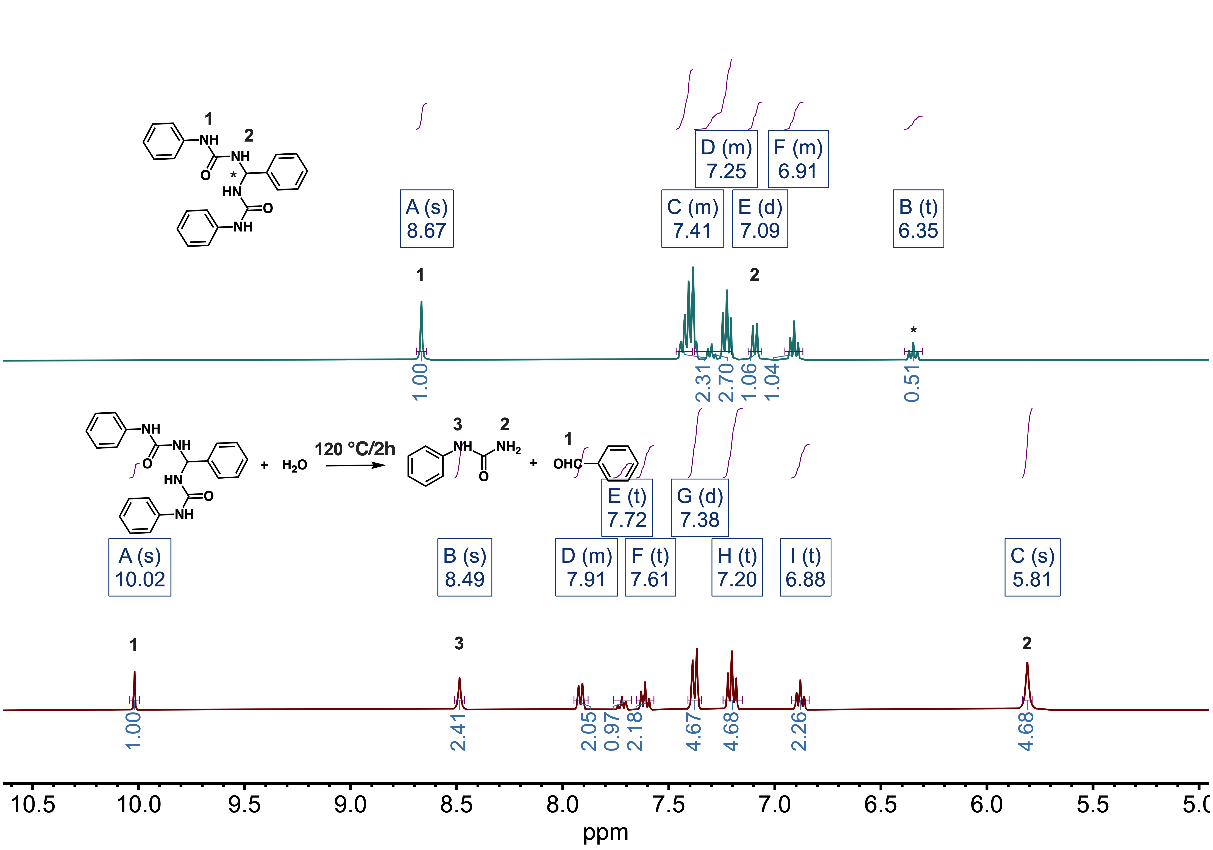
**

Figure S1.^1^H NMR spectra of small molecule 1 and its heating at 120 °C for 2 h.

**
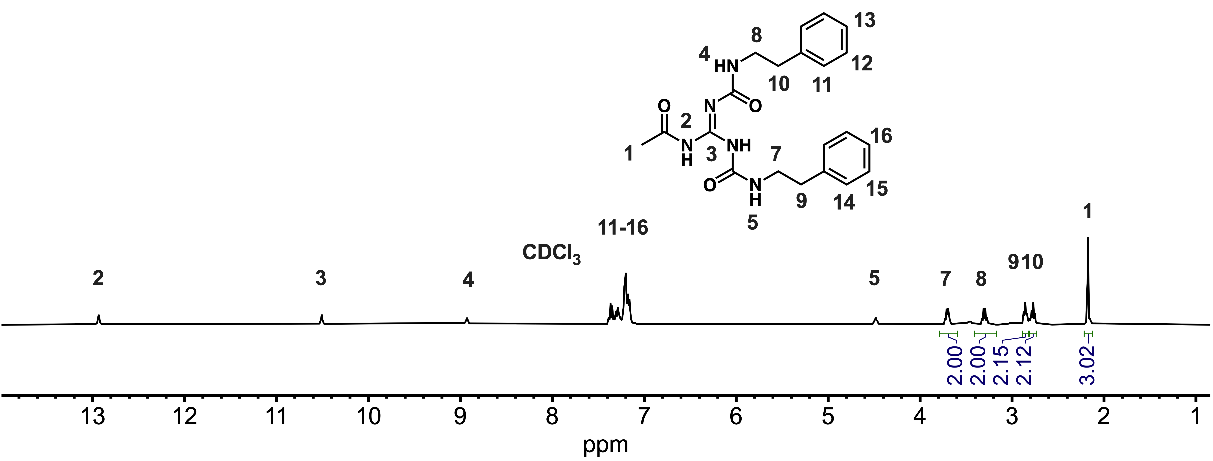
**

Figure S2. ^1^H NMR spectrum of small molecule 2.

**
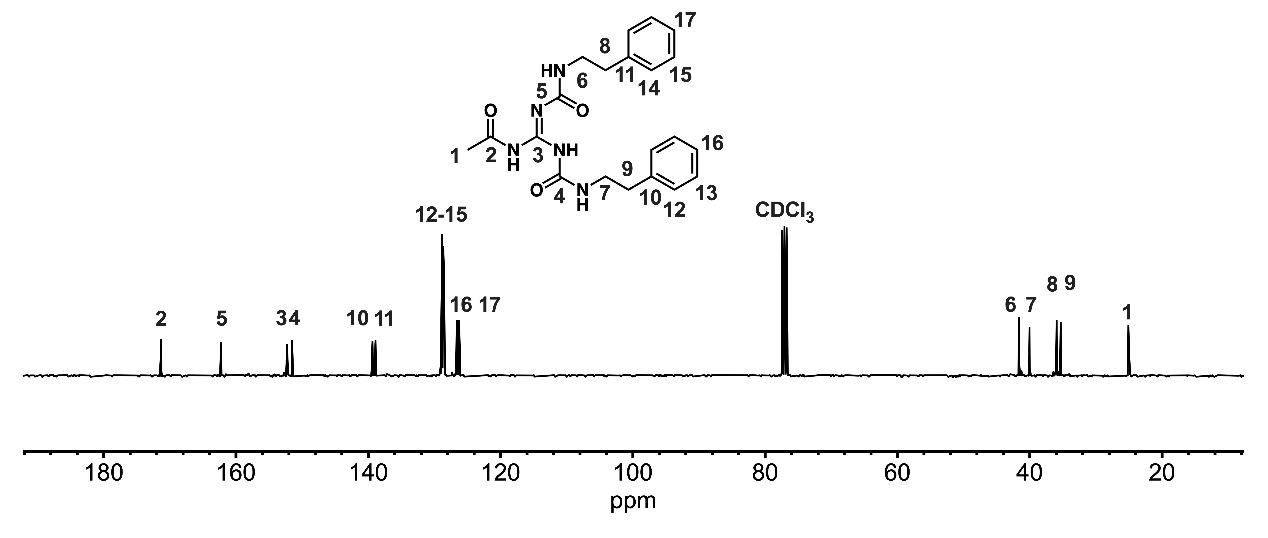
**

Figure S3. ^13^C NMR spectrum of small molecule 2.

**
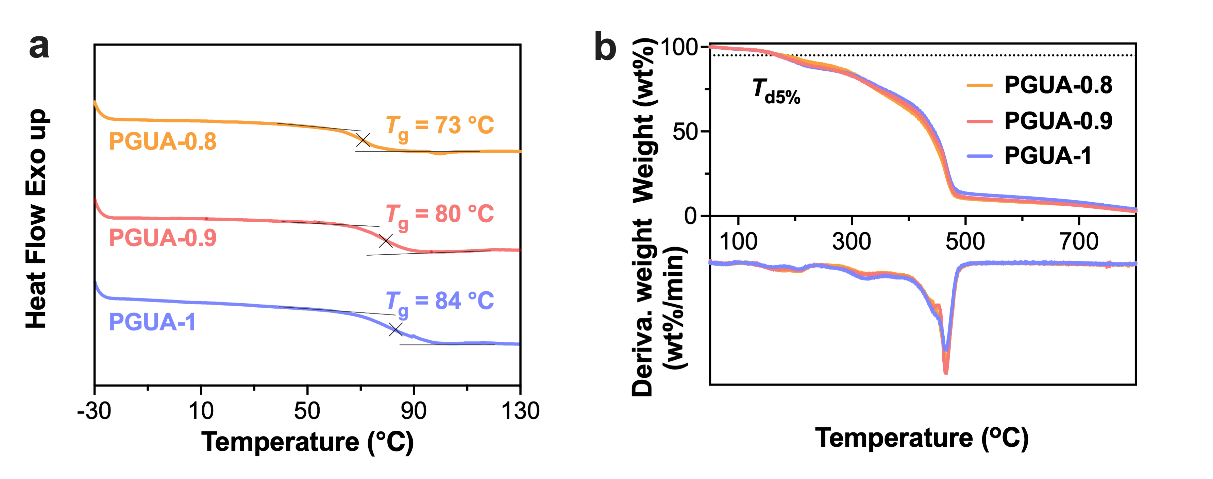
**

Figure S4. (a) To measure the glass transition temperature (*T*_g_), the cured samples were heated from −40 °C to 140 °C at a heating rate of 20 °C min^−1^ and held at 140 °C for 5 min to eliminate thermal history. Then, they were cooled to −40 °C at a cooling rate of 50 °C min^−1^, and heated to 200 °C at a heating rate of 20 °C min^−1^. The *T*_g_ was obtained from the peak temperature of the differential curve of the second heating curve of the cured samples. (b) The thermal stability of samples was recorded at scans ranging from 50 to 800 °C at 10 °C min^-1^ in a N_2_ atmosphere.

**
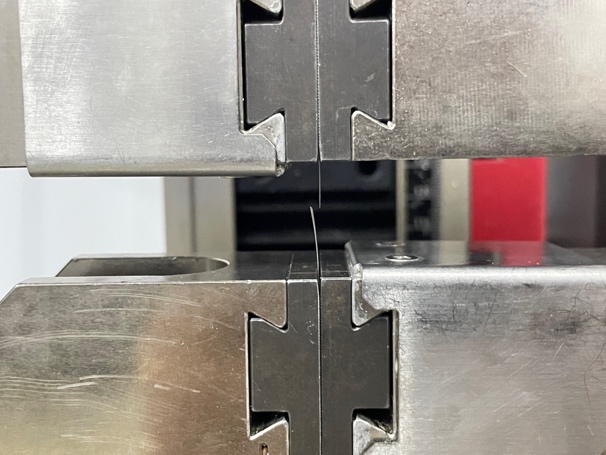
**

Figure S5. The mechanical properties such as tensile strength, Young’s modulus and elongation at break were examined with a universal testing machine. The gauge length was 20 mm, the cross-head speed was 2 mm min^−1^, and the sample size was 40 mm (length) × 5 mm (width) × 0.1 mm (thickness). Above typical photo of PGUA-1 being stretched.

**
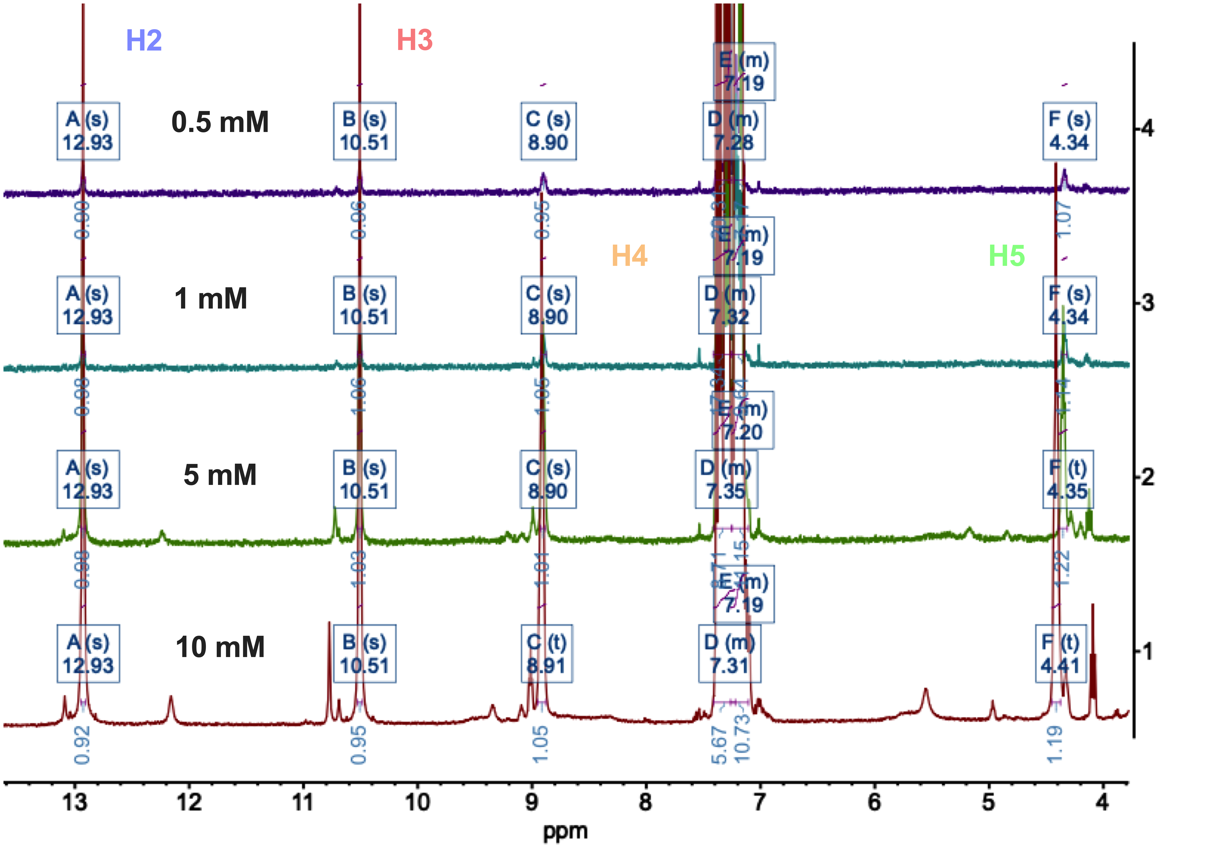
**

Figure S6. GUA intramolecular H-bonding was verified by the *N-H* chemical shift-concentration experiment of small molecule 2. 100 mM sample was prepared by weighing 0.395 g small molecule 2 and 10 mL of CDCl_3_. Dilute 10 times, 20 times, 100 times and 200 times respectively to finally get 10 mM, 5 mM, 1 mM and 0.5 mM samples to be tested via NMR.


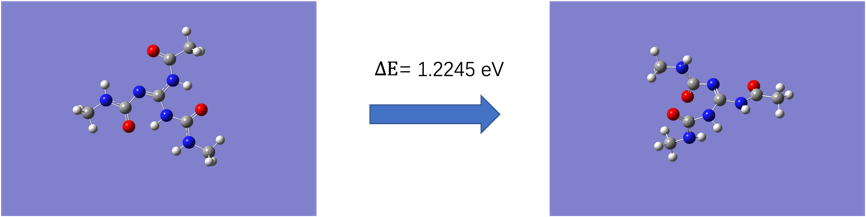


Figure S7. The repeat unit structure of the intramolecular H-bonding was optimized using the density functional theory of the Gaussian 09.D01 program. The B3LYP/6-31G* method was used with the empirical dispersion correction of GD3BJ. The H-bonding was broken through rotation of the torsion around *C-N-C-N*, while the other redundant coordinates were optimized. The highest energy was calculated to be around 30°, and the energy difference with the optimized structure was regarded as the intramolecular H-bond energy. The gray, white, red, and blue spheres represent the C, H, O, and N atoms respectively.


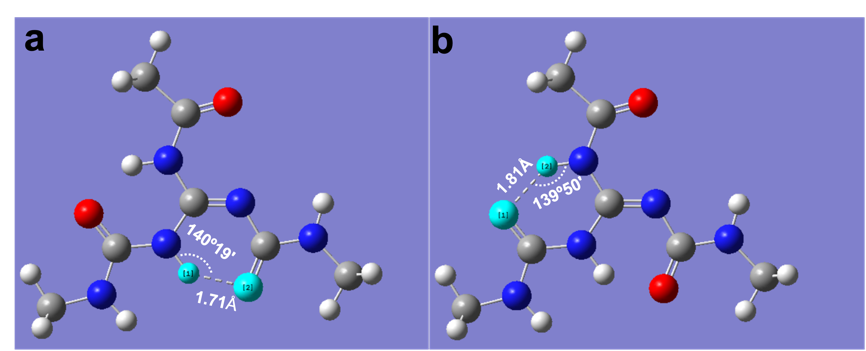


Figure S8. The bonding parameters of GUAs were calculated by DFT theory. The length of the intramolecular H-bonding formed by H3 was 1.71 Å and the bond angle was 140º19'；the bond length and angle by H2 were 1.81 Å and 139º50'.

**
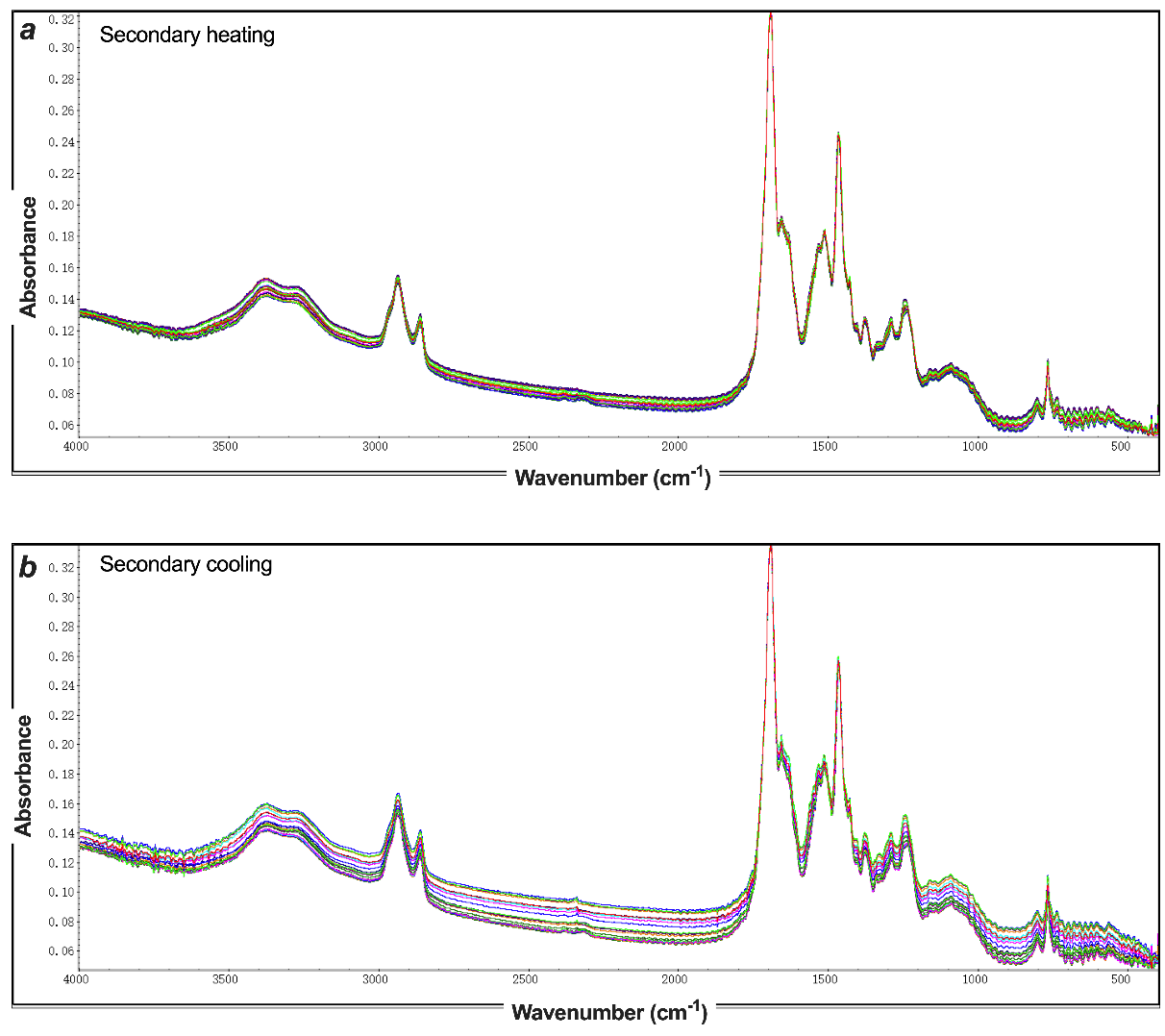
**

Figure S9. *In-situ* FTIR spectra of PGUA-1 was performed using a FTIR instrument with KBr tablet method. The first heating process is used to remove the influence of water peak in the KBr tablet. In-situ FTIR spectra of the second heating and cooling processes were recorded at a range of 30 to 140 °C, a heating rate of 5 °C min^-1^ and staying for 1 minute to collect data.

**
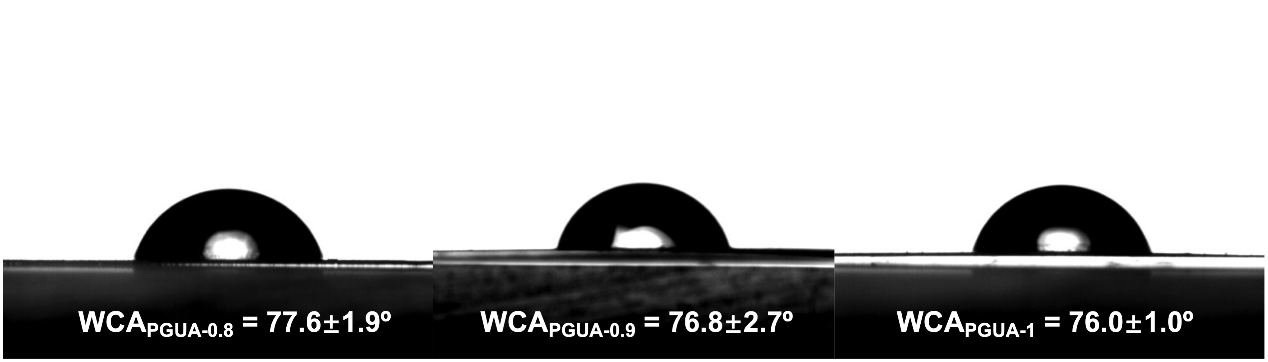
**

Figure S10. Water contact angle (WCA) images of the PGUAs.


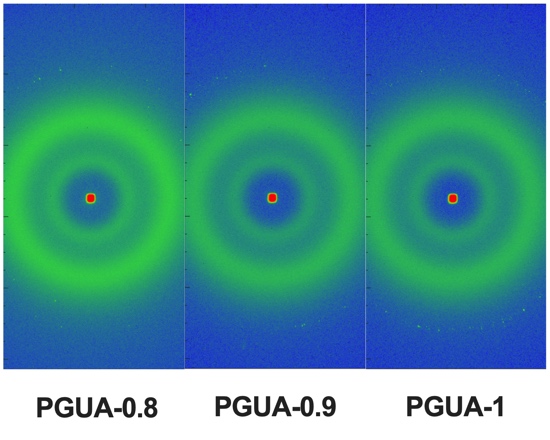


Figure S11. 2D WDXS profiles of PGUAs.


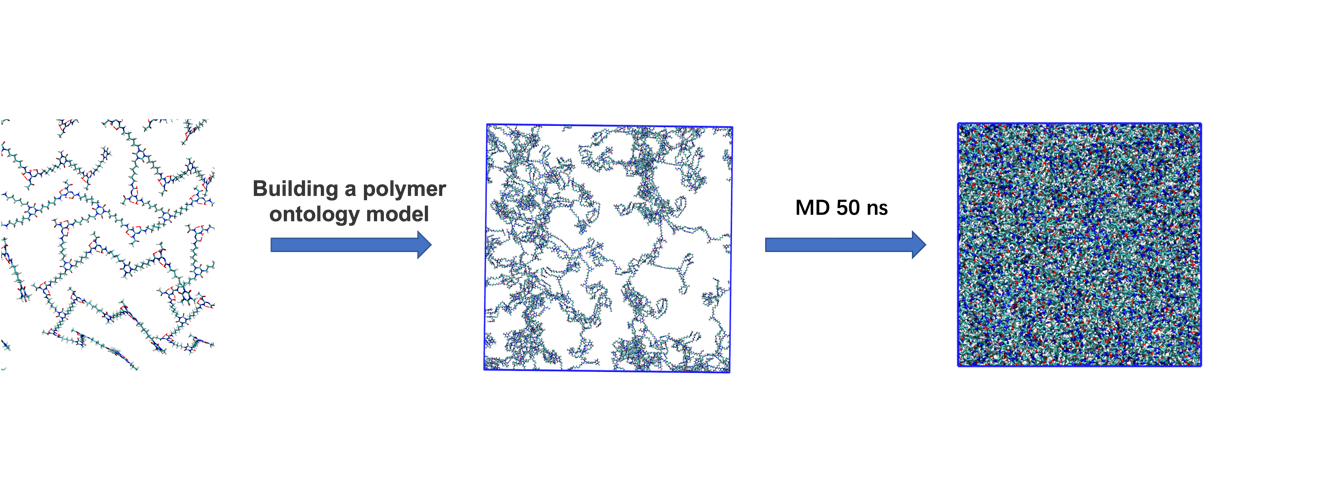


**Figure S12.** Atomistic molecular dynamics simulations have been performed in the GROMACS^[3]^ (version 2020.6) simulation package, using the General Amber force field (GAFF2). The supermolecule polymer with around 2900 atoms was built in the Materials Studio software and 10 molecules were first randomly placed in cubic boxes of around 12 nm. After thousand steps of energy minimization the systems were equilibrated under the NPT ensemble at a relatively elevated temperature of 500 K to relax the polymer structure for 10 ns and then annealed to 298 K within another 10 ns. The production run extended 30 ns and the deformation of the polymer was performed at the rate of 0.5 nm ns^-1^. The temperature was coupled to 298 K using the Nose-Hoover method and the pressure was coupled to 1 atm using the Parrinello-Rahman method. The cutoff scheme of 1.2 nm was implemented for the non-bonded interactions, and the Particle Mesh Ewald method^[4]^ with a fourierspacing of 0.1 nm was applied for the long range electrostatic interactions. All covalent bonds with hydrogen atoms were constraint using the LINCS algorithm^[5]^.

**Molecular dynamics simulation parameters.** The interaction potentials $V$ between atoms include the bonded and non-bonded terms, as **equation S4.**

$V=V_{bonded}+V_{nonbonded}$ (**S4**)

The bonded terms (**equation S5**) include the harmonic oscillation of bonds and angles as well as the torsional rotation of dihedrals; the non-bonded terms (**equation S6**) include the electrostatic interactions between atoms with partial charges as well as the Van der Waals interactions described through the Lennard-Jones 12-6 potentials.

$V_{bonded}=\sum_{bonds} \frac{1}{2}k_{b}\left( l-l_{0} \right)^{2}+\sum_{angles} \frac{1}{2}{k_{\theta}\left( \theta-\theta_{0} \right)}^{2}+\sum_{torsions} k_{\phi}\left[ 1+cos\left( n\phi-\phi_{0} \right) \right]$ (**S5**)

$V_{nonbonded}=\sum_{i=1}^{N} \sum_{j=i+1}^{N} \left\{ \frac{q_{i}q_{i}}{4\pi\epsilon_{0}\epsilon_{r}r_{ij}}+4\varepsilon_{ij}\left[ \left( \frac{\sigma_{ij}}{r_{ij}} \right)^{12}-\left( \frac{\sigma_{ij}}{r_{ij}} \right)^{6} \right] \right\}$ (**S6**)

The General Amber force field (GAFF2) was used and the Lennard-Jones parameters are listed in the table below (hc, c3, n, h1, c, hn, o, c2, n2 were atomic type in force field):

| $\boldsymbol{V}_{\boldsymbol{nonbonded}}$ | $\boldsymbol{\varepsilon}_{\boldsymbol{ij}}$  (kJ/mol) | $\boldsymbol{\sigma}_{\boldsymbol{ij}}$  (nm) |
| --- | --- | --- |
| hc | 6.568880E-02 | 2.649533E-01 |
| c3 | 4.577296E-01 | 3.399670E-01 |
| n | 7.112800E-01 | 3.249999E-01 |
| h1 | 6.568880E-02 | 2.471353E-01 |
| c | 3.598240E-01 | 3.399670E-01 |
| hn | 6.568880E-02 | 1.069078E-01 |
| o | 8.786400E-01 | 2.959922E-01 |
| c2 | 3.598240E-01 | 3.399670E-01 |
| n2 | 7.112800E-01 | 3.249999E-01 |


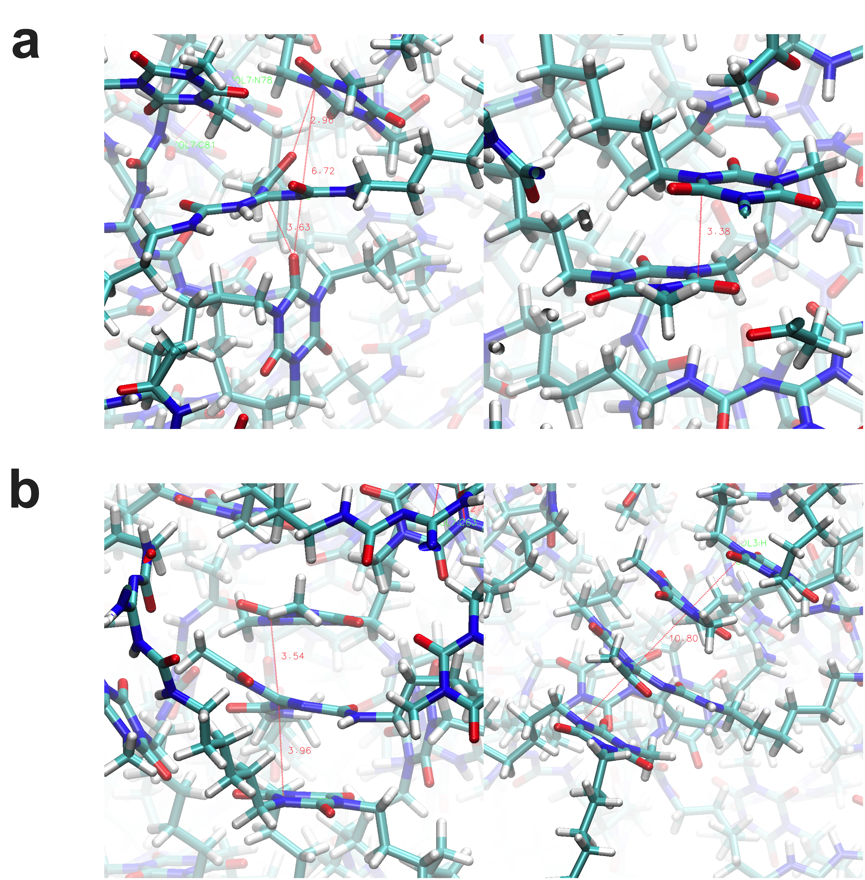


Figure S13. In GUA-SPs network, the forming of π-π stacking (including face-to-face and edge-to-face) and the operating distance are obtained by MD simulation.

**
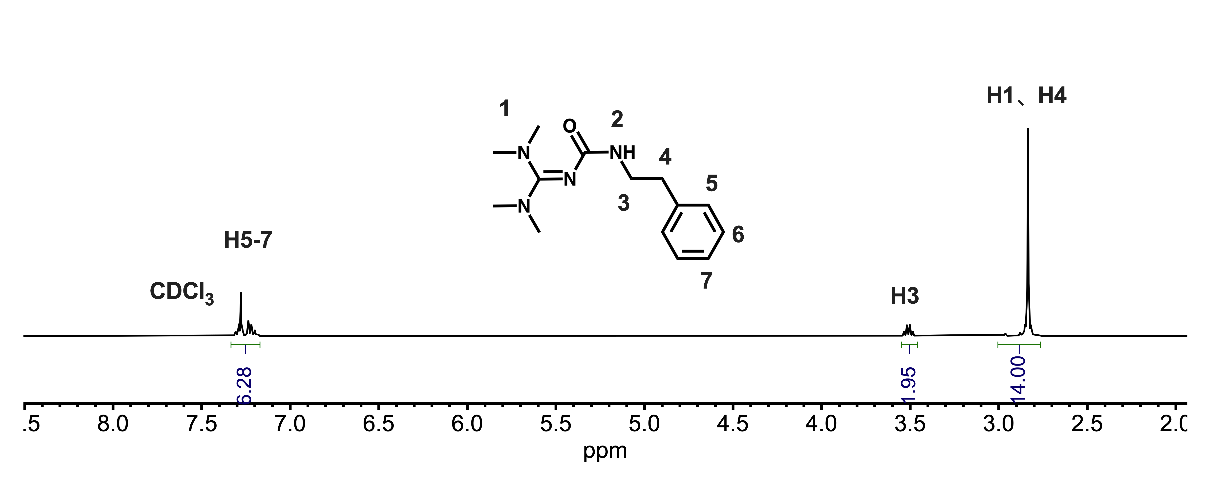
**

Figure S14.^1^H NMR spectrum of small molecule 3.

**
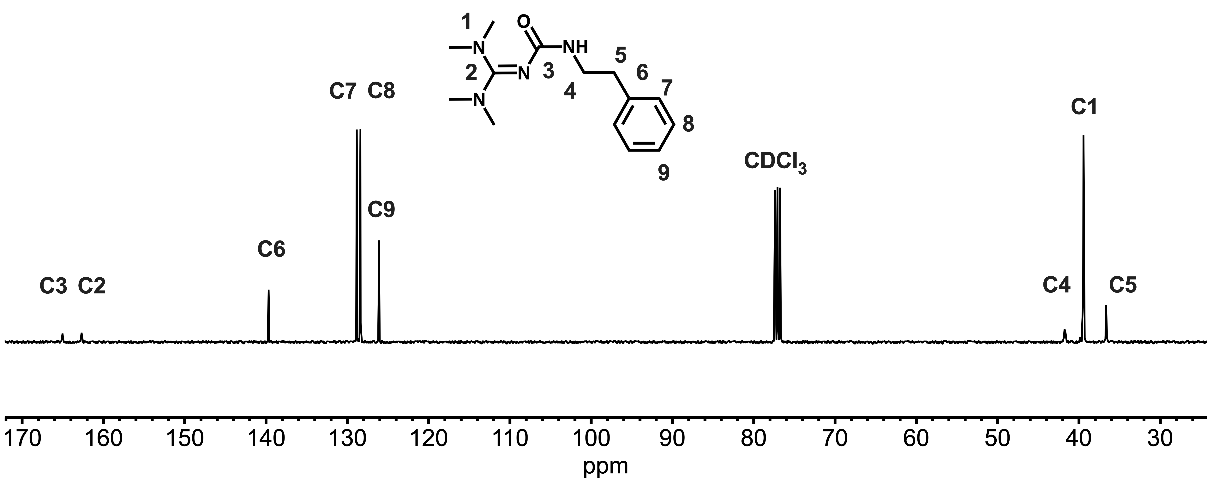
**

Figure S15. ^13^C NMR spectrum of small molecule 3.

**
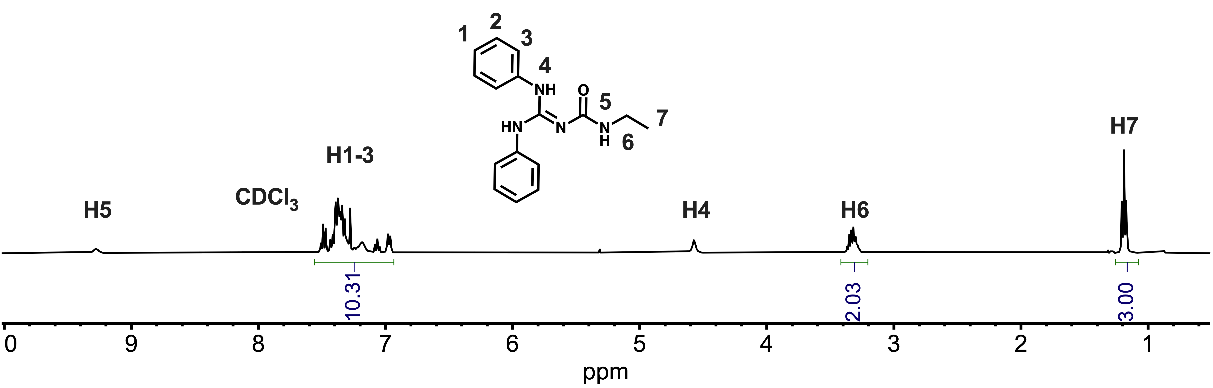
**

Figure S16. ^1^H NMR spectrum of small molecule 4.

**
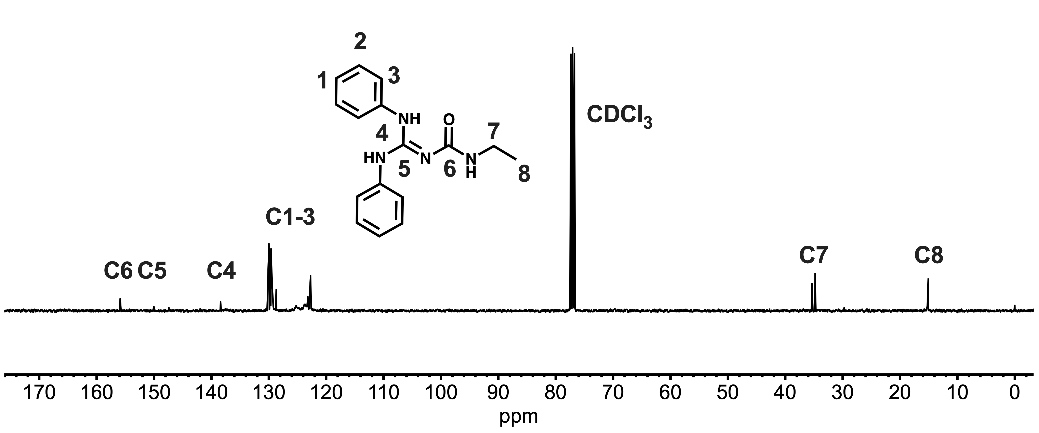
**

Figure S17. ^13^C NMR spectrum of small molecule 4.

**
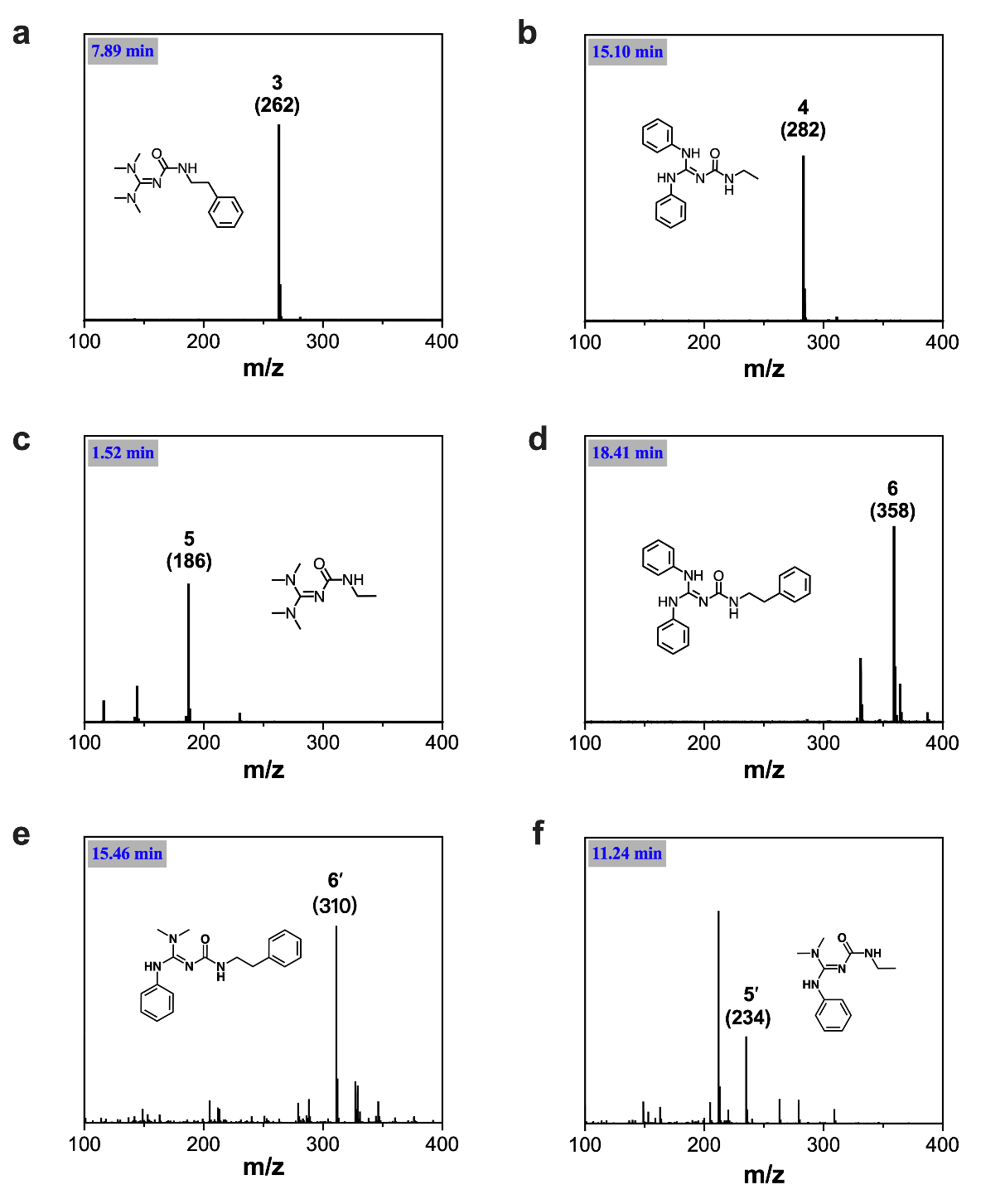
**

Figure S18. Mass spectra of model 3, 4, 5, 6, 5’ and 6’ separated by liquid chromatograph.


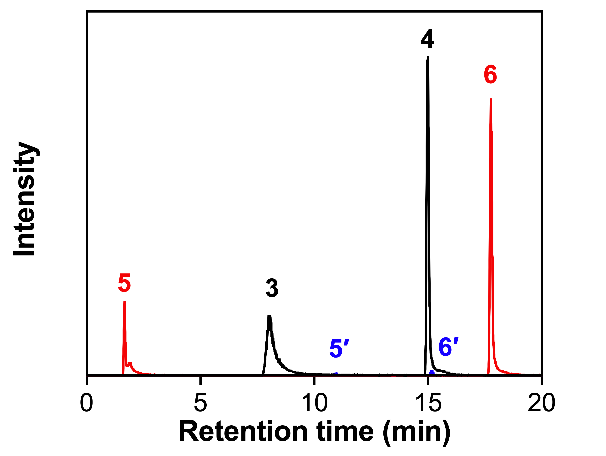


Figure S19. LC-MS spectra of all models separated by liquid chromatograph after reaction at 140 °C for 1 h.


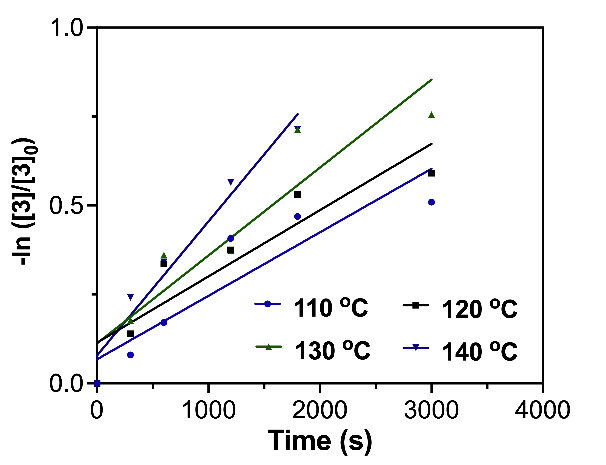


Figure S20. Plots of $\boldsymbol{-}\mathbf{ln}\boldsymbol{([3]}\boldsymbol{[3]}_{\boldsymbol{0}}\boldsymbol{)}$ versus time (*t*) for model 3. *k* at each temperature was determined by its slope value. This reaction can be described in terms of pseudo-first kinetics (equation S7).^[6]^

${d[3]}/{dt=-k[3]}$ (**S7**)

If the initial concentration of 3 is described as [3]_0_, **equation S7** can be transformed to **equation S8.**

$\ln\left( \left[ 3 \right]/{\left[ 3 \right]_{0}} \right)=-kt$ (**S8**)

110 °C y = 0.0001785x + 0.06773 R^2^=0.8409

120 °C y = 0.0001863x + 0.11450 R^2^=0.8403

130 °C y = 0.0002470x + 0.11250 R^2^=0.8666

140 °C y = 0.0003770x + 0.07817 R^2^=0.9578

To calculate the activation energy of each reaction, the Arrhenius diagram was plotted using *k*_exp_ (**equation S9**).

$k=-Aexp(-{E_{a}}/{RT})$ (**S9**)

where *A* is the Arrhenius pre-factor, *E*_a_ is activation energy, *R* is the gas constant, and *T* is the temperature.

**
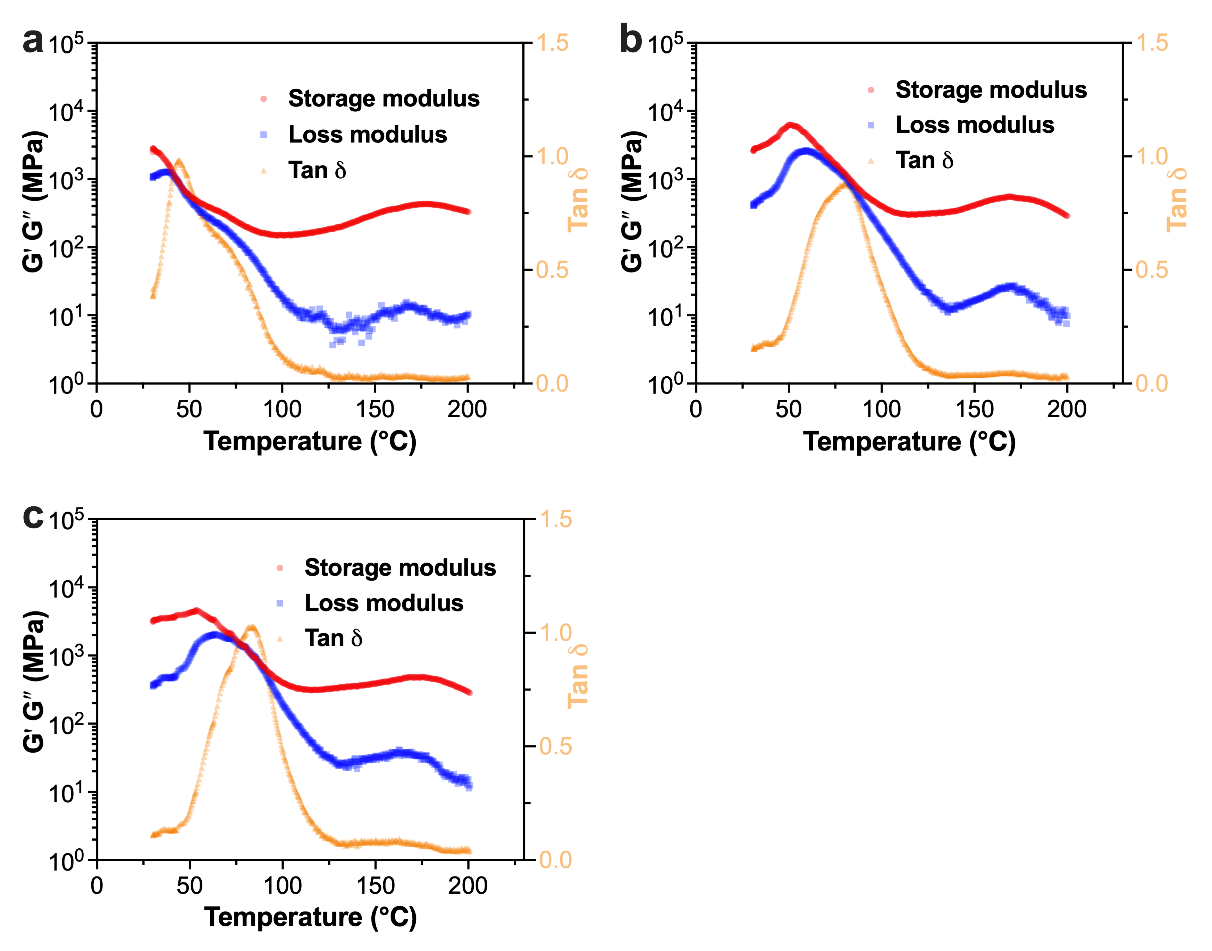
**

Figure S21. The rheological behavior of all samples was recorded from 30 to 200 °C at 5 °C min^-1^ with an axial force fixed at 2 N in a N_2_ atmosphere.


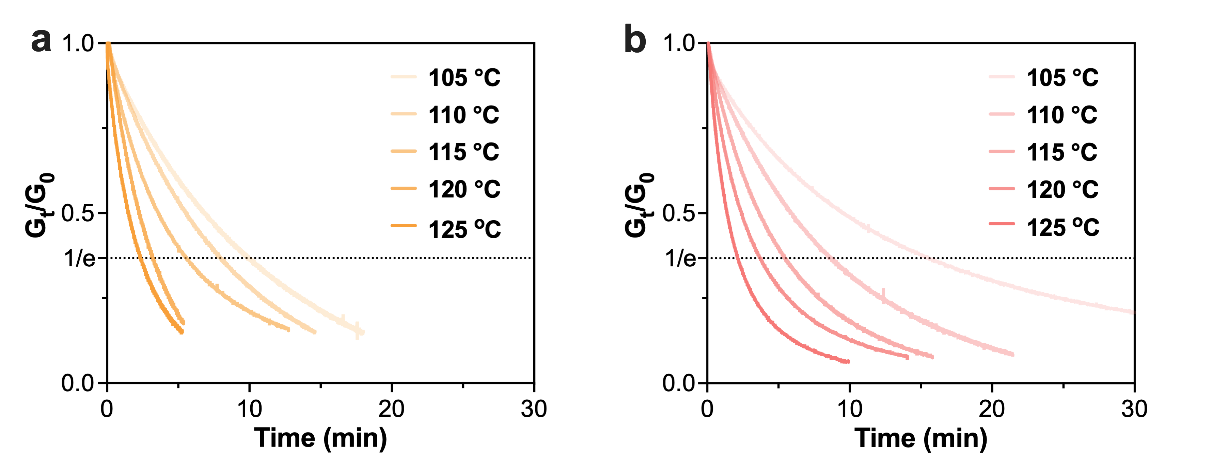


Figure S22. Stress relaxation curves of PGUA-0.8 and PGUA-0.9 at varying temperatures. Stress relaxation experiments were carried out using Q850 DMA. All samples with dimensions of 20 mm (length) × 5 mm (width) × 0.1 mm (thickness) were by a 3% constant strain and a 0.005 N preload. When the test temperature was attained, it was maintained for 3 min to reach thermal equilibrium. The relaxation time *τ** obeyed the Arrhenius equation S10 with the temperatures for all samples, which can be adopted to estimate the *E*_a_s of the bond-exchange reaction.^[7]^

$\tau^{*}\left( T \right)=\tau_{0}^{*}e^{{E_{a}}/{RT}}$ (**S10**)

where *τ*_0_* is the Arrhenius pre-factor, *R* is the gas constant, and *T* is the temperature.


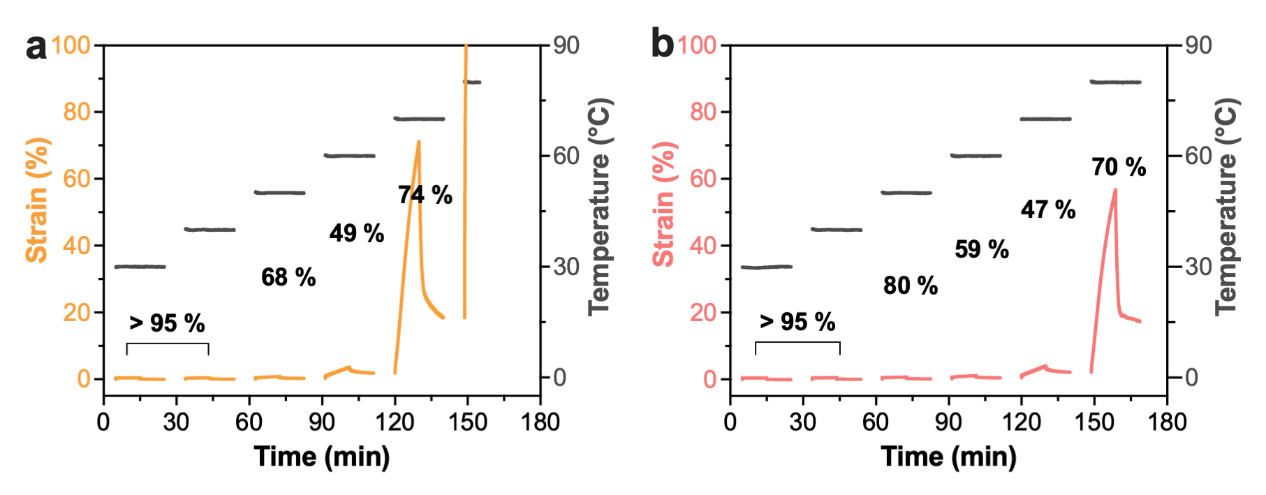


Figure S23. TTS creep curves for PGUA-0.8 (a) and PGUA-0.9 (b). TTS creep experiments were also carried out using Q850 DMA. All PGUAs with dimensions of 20 mm (length) × 5 mm (width) × 0.1 mm (thickness) were by a 5 MPa stress for 10 min and then removed and recovered at varying temperatures for 10 min in a stage of 10 °C starting at 30 °C.


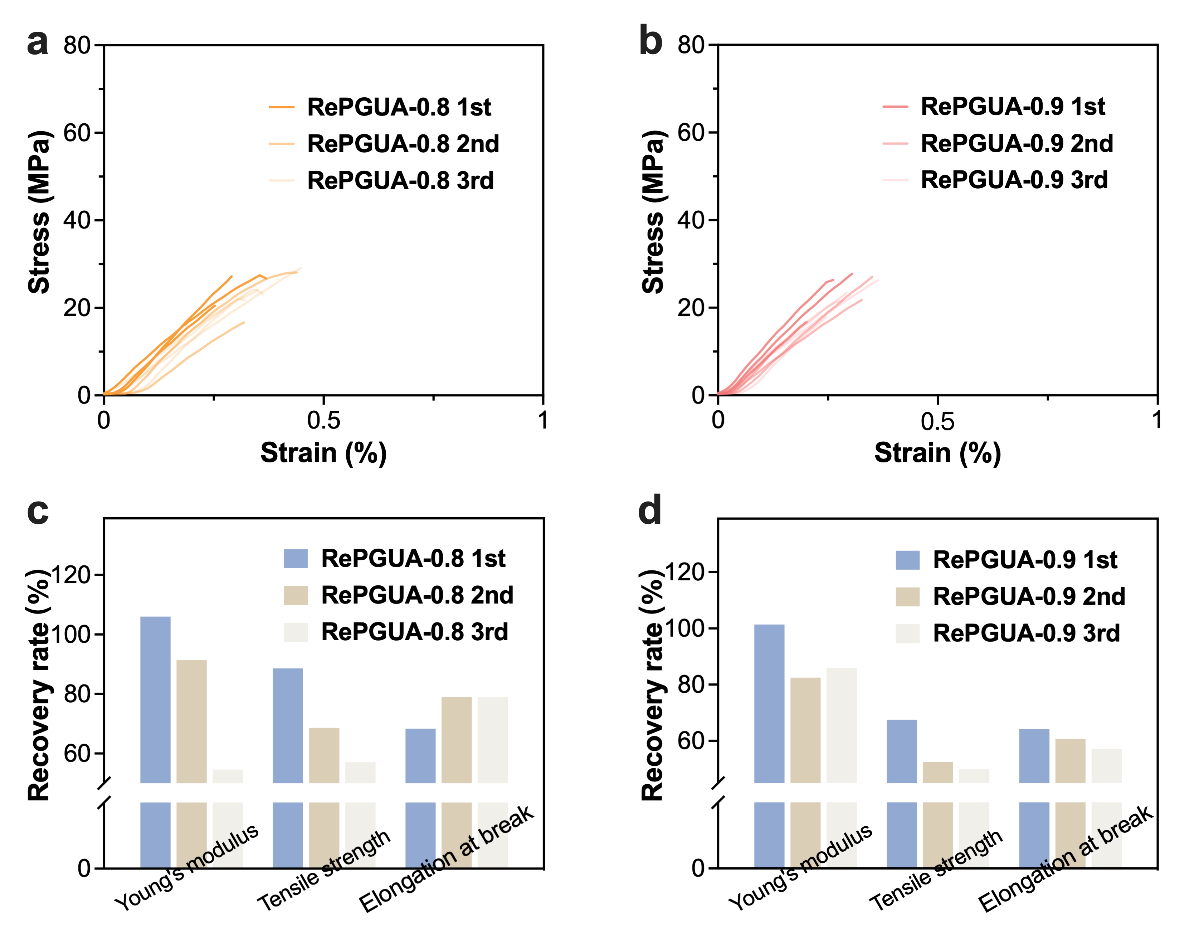


Figure S24. Stress-strain curves (a-b) of the reprocessed PGUA-0.8, PGUA-0.9 and its recovery rate of mechanical properties (c-d).


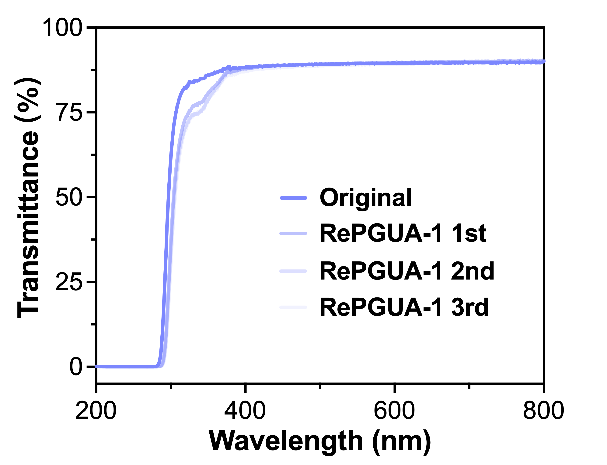


Figure S25. UV-vis transmission spectra of the reprocessed PGUA-1.


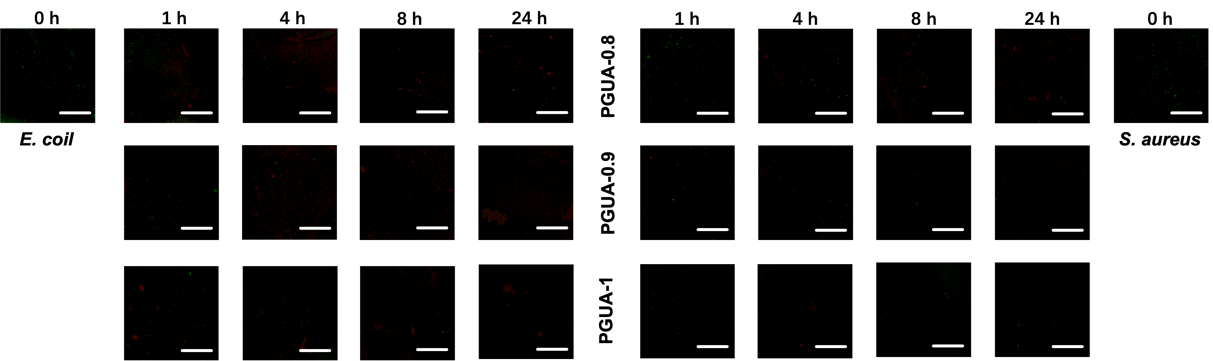


Figure S26. The live/dead staining of PGUAs at 0 h, 1 h, 4 h, 8 h and 24 h for inhibition of *E. coil* and *S. aureus*. The unmarked scale bars represent 500 μm.


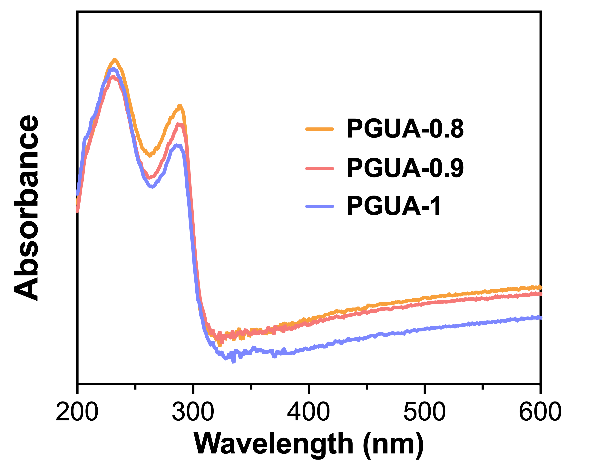


Figure S27. UV absorption spectra of all PGUAs.


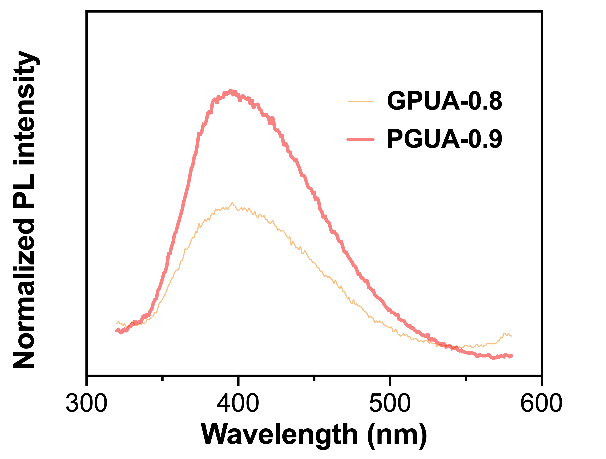


Figure S28. Fluorescence emission spectra of PGUA-0.8 and PGUA-0.9.


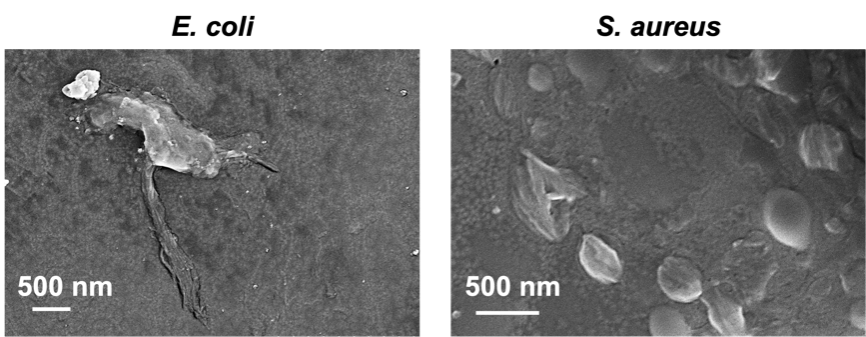


Figure S29. Morphology of *E. coli* and *S. aureus* on PGUA-1 for 1 h under light.


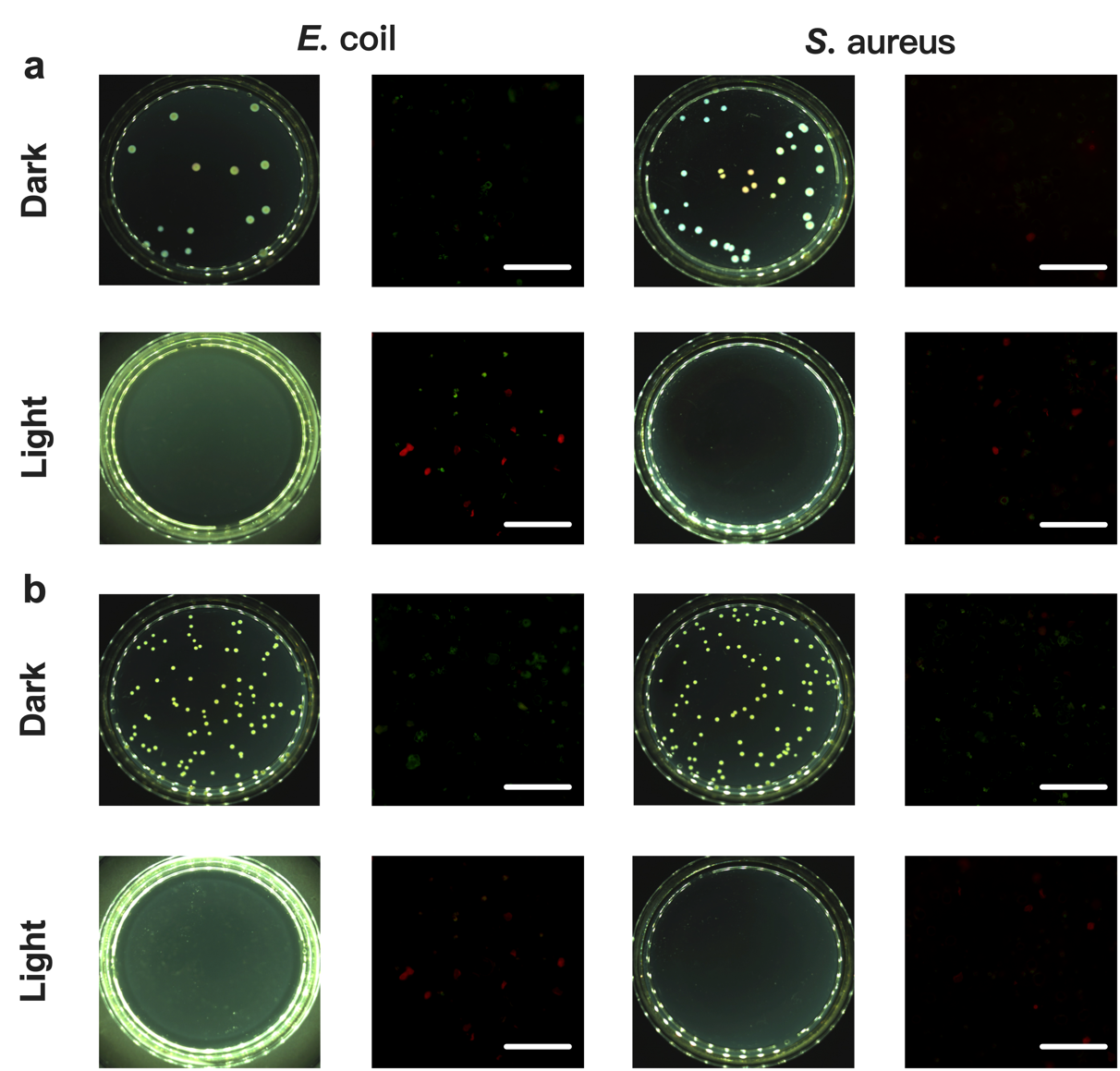


Figure S30. Coating plate pictures and live/dead double staining images on *E. coli* and *S. aureus* under dark and light conditions of 1 h each after treated with coated PGUA-0.8 and PGUA-0.9 films. The unmarked scale bars represent 500 μm.


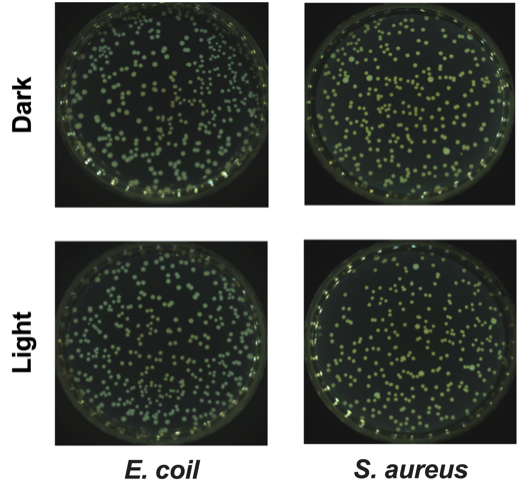


Figure S31. The photos of *E. coil* and *S. aureus* coated plates in light or dark incubator for 1h.


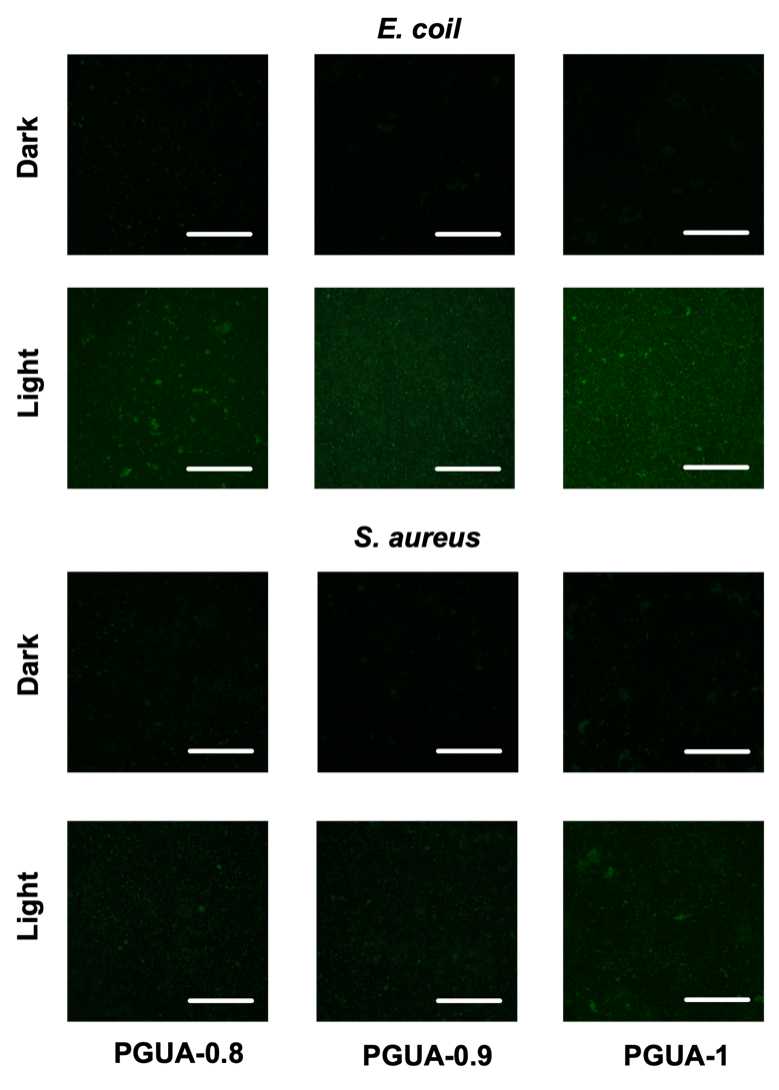


Figure S32. Fluorescence images of *E. coli* and *S.aureus* incubated with PGUAs for 1 h under dark or light conditions. The green areas are the substances that DCFH-DA is oxidized to by ROS. The unmarked scale bars represent 500 μm.


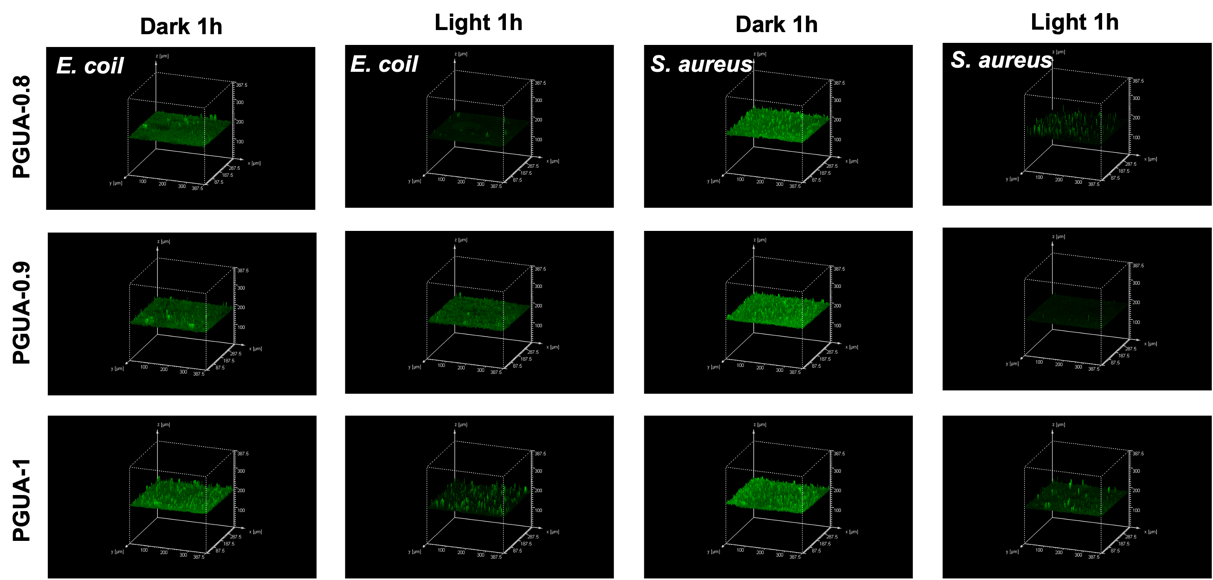


Figure S33. Representative laser scanning confocal 3D images of biofilms under different conditions.


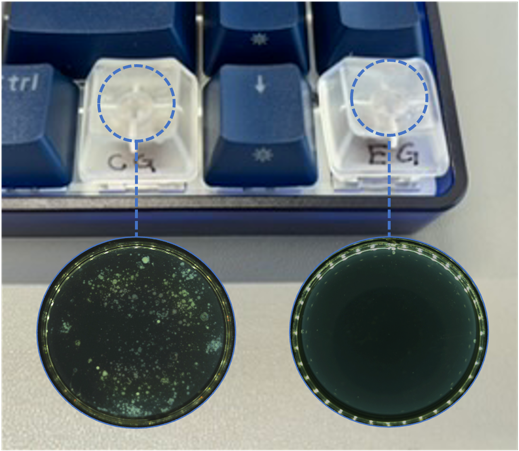


Figure S34. Photos of PGUA-1 as a plastic keyboard key surface material and its antibacterial activity after two weeks of use. CG is the control group, and EG is the experimental group.


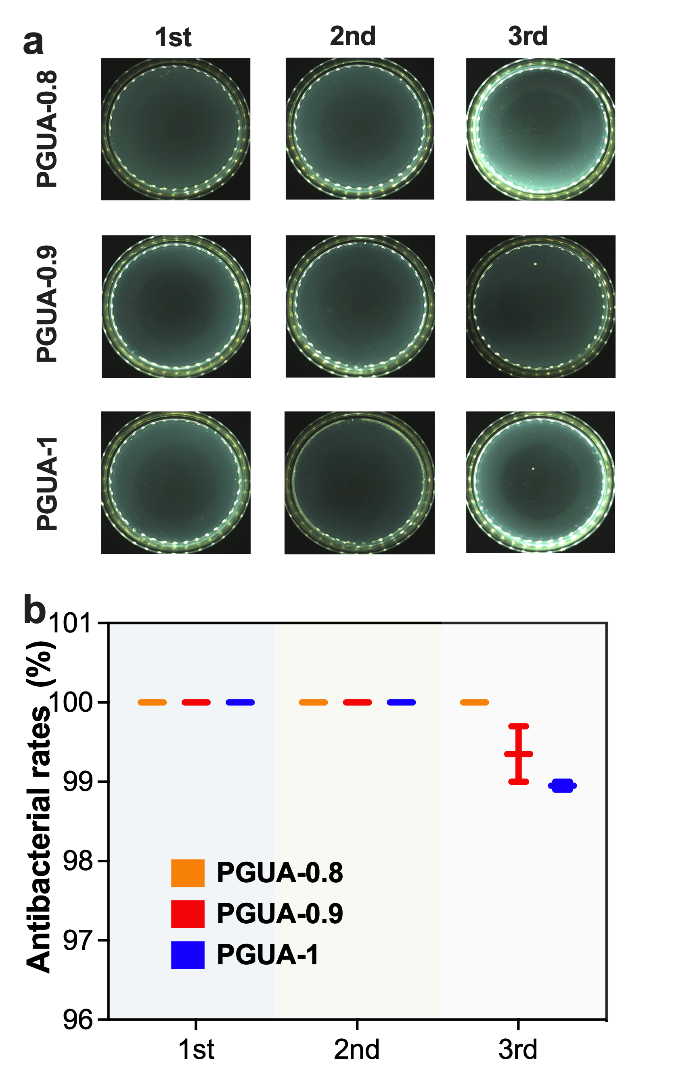


Figure S35. (a) The digital photos of the reprocessed PGUAs on *E. coil* at 24 h by the spread plate method; (b) Antibacterial rate of the reprocessed PGUAs.


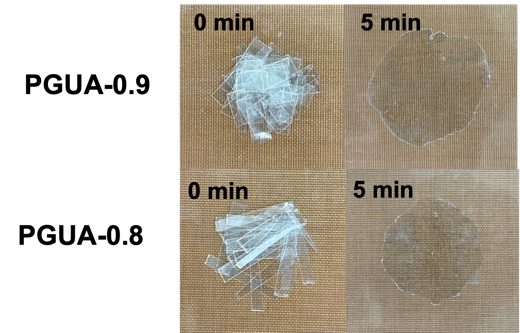


Figure S36. Digital photos of samples PGUA-0.9 and PGUA-0.8 before and after reprocessing with 10 MPa for 5 min at 140 °C.

1. **Supplementary Tables**

**Table S1.** Formulations of PGUAs, the mass fraction of GUAs, and the N element content (XPS) of all networks.

| **Sample** | **compositions (g)** | | | **GUAs (%)** | **N (%)** |
| --- | --- | --- | --- | --- | --- |
|  | AG | D230 | tri-HDI |  |  |
| PGUA-0.8 | 0.121 | 0.069 | 0.529 | 18.7 | 4.58 |
| PGUA-0.9 | 0.136 | 0.035 |  | 21.5 | 5.91 |
| PGUA-1 | 0.152 | 0 |  | 24.8 | 7.22 |

**Tables S2.** Partial physical properties and recovery rates of the reprocessed PGUAs.

| **Samples** | **Young’s modulus (𝛦, GPa)** | **Recovery rate of 𝜎** | **Tensile strength (𝜎, MPa)** | **Recovery rate of 𝛦** | **Elongation at break (𝜀, %)** | **Recovery rate of 𝜀** | **Transmittance (T %)** | **Recovery rate of T%** |
| --- | --- | --- | --- | --- | --- | --- | --- | --- |
| PGUA-0.8 | 8.801 ± 2.048 | - | 35 ± 3 | - | 0.57 ± 0.06 | - | 91.58 | - |
| RePGUA-0.8 1st | 9.322 ± 0. 652 | 106.0% | 31 ± 6 | 88.6% | 0.39 ± 0.04 | 68.4% | - | - |
| RePGUA-0.8 2nd | 8.046 ± 0.562 | 91.4% | 24 ± 4 | 68.6% | 0.45 ± 0.18 | 78.9% | - | - |
| RePGUA-0.8 3rd | 4.805 ± 1.014 | 54.6% | 20 ± 4 | 57.1% | 0.45 ± 0.12 | 78.9% | - | - |
| PGUA-0.9 | 9.521 ± 0.909 | - | 40 ± 7 | - | 0.56 ± 0.10 | - | 91.38 | - |
| RePGUA-0.9 1st | 9.657 ± 1.652 | 101.4% | 27 ± 1 | 67.5% | 0.36 ± 0.05 | 64.3% | - | - |
| RePGUA-0.9 2nd | 7.859 ± 0.959 | 82.5% | 21 ± 5 | 52.5% | 0.34 ± 0.08 | 60.7% | - | - |
| RePGUA-0.9 3rd | 8.182 ± 0.792 | 85.9% | 20 ± 3 | 50.0% | 0.32 ± 0.02 | 57.1% | - | - |
| PGUA-1 | 14.037 ± 0.850 | - | 60 ± 4 | - | 0.53 ± 0.05 | - | 90.78 | - |
| RePGUA-1 1st | 11.662 ± 1.317 | 83.1% | 49 ± 4 | 81.7% | 0.47± 0.12 | 88.7% | 90.92 | 100.2% |
| RePGUA-1 2nd | 10.598 ± 1.686 | 75.5% | 44 ± 7 | 73.3% | 0.55 ± 0.05 | 103.8% | 91.17 | 100.4% |
| RePGUA-1 3rd | 10.020 ± 0.835 | 71.4% | 38 ± 7 | 63.3% | 0.48 ± 0.09 | 90.6% | 90.74 | 100% |

1. **Supplementary References**

[1] a)M. Yusuf, S. Thakur, *Journal of Heterocyclic Chemistry* **2019**, 56, 3403; b)D. Fu, W. Pu, J. Escorihuela, X. Wang, Z. Wang, S. Chen, S. Sun, S. Wang, H. Zuilhof, H. Xia, *Macromolecules* **2020**, 53, 7914.

[2] a)L. W. Hill, *Prog. Org. Coat.* **1997**, 31, 235; b)J. Scanlan, *Journal of Polymer Science Part A: Polymer Chemistry* **1960**, 43, 501.

[3] B. Hess, C. Kutzner, D. van der Spoel, E. Lindahl, *J. Chem. Theory Comp.* **2008**, 4, 435.

[4] U. Essmann, L. Perera, M. L. Berkowitz, T. Darden, H. Lee, L. G. Pedersen, *J. Chem. Phys.* **1995**, 103, 8577.

[5] B. Hess, H. Bekker, H. J. C. Berendsen, J. G. E. M. Fraaije, *J. Comput. Chem.* **1997**, 18, 1463.

[6] Q. Li, S. Ma, P. Li, B. Wang, H. Feng, N. Lu, S. Wang, Y. Liu, X. Xu, J. Zhu, *Macromolecules* **2021**, 54, 1742.

[7] M. Capelot, M. M. Unterlass, F. i. Tournilhac, L. Leibler, *ACS Macro Lett.* **2012**, 1, 789.
